# AutoTFCNNY: A multi-instance neural network for enhanced early cancer detection using TCR data

**DOI:** 10.1371/journal.pone.0326253

**Published:** 2025-10-08

**Authors:** Donghong Yang, Xin Peng, Yiming Zhou, Shenglan Peng

**Affiliations:** 1 Jingdezhen Ceramic University, Jingdezhen, China; 2 Base and Byte Biotechnology Company Ltd., Beijing, China; University of Jaen: Universidad de Jaen, SPAIN

## Abstract

For most cancers, early diagnosis and intervention can significantly improve cure rates and patient survival. Consequently, achieving early and accurate cancer detection has always been a central focus in both medical practice and scientific research. Recently, studies based on peripheral blood T-cell receptors (TCRs) have attracted considerable attention due to their noninvasiveness and potential for high sensitivity. It has been reported that cancer-associated TCRs (caTCRs) exist in the peripheral blood of cancer patients, suggesting that discerning whether a TCR repertoire is associated with cancer provides a viable strategy for early cancer prediction. However, extracting crucial cancer-related information from a large and heterogeneous TCR repertoire remains a major challenge.To address this issue, we propose AutoTFCNNY, a multi-instance deep neural network model that combines a Transformer and a convolutional neural network (CNN). Built upon a multi-instance learning (MIL) framework, AutoTFCNNY leverages the Transformer’s global dependency modeling alongside the CNN’s local feature enhancement to effectively extract TCR sequence features, thereby significantly improving early cancer detection accuracy. Experimental results demonstrate that AutoTFCNNY performs well in detecting 22 different cancer types, achieving an average area under the ROC curve (AUC) exceeding 0.94. Notably, in 18 of these types—including brain cancer and non-small cell lung cancer et al.—the average AUC surpasses 0.99. These findings indicate that AutoTFCNNY possesses high accuracy, stability, and favorable generalization ability, suggesting its potential as a non-invasive tool for early cancer detection based on peripheral blood TCR repertoires.

## Introduction

In previous studies, a variety of assays have been widely used for limited types of early cancer detection, including medical imaging [1,2], gene expression analysis [3–6], identification of single nucleotide polymorphisms (SNPs) [7–9], detection of tumor biomarkers (e.g., prostate-specific antigen, CA-125, and CA-153) [10,11], and single-cell and high-throughput sequencing technologies (e.g., cell-free DNA (cfDNA) and circulating tumor cells in liquid biopsies) [12,14], among others. In recent years, deep learning-driven medical image analysis has also made significant advances. Models such as SNC_Net, DVFNet, and SCDNet have substantially improved the automatic diagnostic accuracy for various types of skin cancer and other tumors, while federated learning, big data, and Internet of Things (IoT) technologies have greatly facilitated the deployment of medical AI in multi-center scenarios [15–20]. These approaches provide valuable references in terms of automation, feature fusion, and model generalization. However, whether traditional molecular detection methods or emerging AI-based imaging approaches, most still rely on visible tumors or specific molecular markers. For certain early-stage and occult cancers, both sensitivity and specificity remain limited, and the high cost of detection restricts their utility in large-scale screening programs.

Recently, cancer diagnostic models related to the immune system, especially those based on T cell receptor (TCR) repertoire data, have attracted increasing attention due to their non-invasiveness and potential for early detection. T cells play a crucial role in anti-tumor immune responses, and their surface TCRs are specialized in recognizing antigens [21–23]. During tumor progression, the T cell repertoire often undergoes cancer-specific changes [24], and these TCRs are defined as cancer-associated TCRs (caTCRs). Studies have shown that certain caTCRs may share common biochemical features [25,26]. Therefore, distinguishing the TCRs of healthy individuals from those of cancer patients has become an important research topic, particularly focusing on the CDR3 region of the TCR*β* chain, which is of great significance for discriminating between healthy and cancerous individuals [27].

Although the precise biochemical properties of caTCRs are still being explored, the development of adaptive immune receptor repertoire sequencing (AIRR-SEQ) has significantly changed our understanding of TCR repertoire at the individual and population levels and generated a large amount of sequencing data [28]. Using this sequencing data, many deep learning frameworks for detecting caTCRs have been developed. These computational frameworks hold promising potential in early cancer screening, as well as in predicting cancer immune responses and the effectiveness of immunotherapy. Among these approaches, Beshnova et al. [29] developed a deep learning-based model named DeepCAT, which achieved de novo prediction of caTCR. DeepCAT demonstrated superior accuracy in caTCR prediction and TCR repertoire classification, mainly due to the clustering strategy employed during preprocessing and the strong feature extraction capabilities of deep learning. However, DeepCAT overlooks the correlations among TCRs within the same repertoire by assigning identical weights to all TCRs in that repertoire. Since these TCRs may have varying contributions, they should potentially receive equal weights.Additionally, when using the TCR repertoire to predict a patient’s tumor status, multiple distinct TCR sequences (instances) may be observed in different T cells of the same patient (whether tumor or normal). Therefore, this prediction problem can be classified within the framework of MIL. Therefore, Xiong et al. [30] explored the application of MIL methods using TCR sequences for cancer detection. The study focused on evaluating the applicability of 16 different MIL methods in cancer detection. Experimental results showed that, with the appropriate MIL method, satisfactory performance was achieved in five out of ten types of cancer, with the area under the receiver operating characteristic (ROC) curve exceeding 80%. Ostmeyer et al. [31] attempted to model TCR correlations using Multi-Instance Learning. However, predicting cancer solely based on the presence of an anomalous TCR in a repertoire is not appropriate and increases the risk of false positives, as cancer patients’ repertoires typically contain multiple correlated caTCRs. However, the aforementioned caTCR prediction methods do not adequately account for cancer-associated biochemical motifs of varying lengths and exhibit shortcomings in modeling the correlations among TCRs within the same repertoire. Consequently, the DeepLION model proposed by Xu et al. [32] employs a multi-instance learning approach to consider the correlations between TCRs of different lengths and utilizes alternating convolutional filters along with 1-max pooling operations to process amino acid fragments, thereby further enhancing prediction accuracy. Kim et al. [33] proposed a Multi-Instance Neural Network model, MINN-SA, based on a sparse attention mechanism. This model uses the sparsemax function to sparsify the attention weights, enabling it to adaptively focus on key instances within the samples. MINN-SA performed remarkably well on various tumor-type datasets, surpassing both traditional machine learning and other deep learning methods in accuracy and efficiency. Qian et al. [34] introduced DeepLION2 as an enhancement of the original DeepLION model. DeepLION2 effectively captures the correlation between TCRs by incorporating a sparse self-attention mechanism, enabling it to focus on the most relevant TCRs for each individual TCR. Furthermore, this framework leverages a contrastive learning strategy to update the attention matrix during training, preventing the model from emphasizing TCRs unrelated to cancer. Recently, Yideng et al. [35] successfully constructed a deep learning framework named iCanTCR by integrating CNN and LSTM, aiming to capture critical features in the TCR sequences of cancer patients. Additionally, an abundance weighting strategy was introduced to highlight high-abundance TCRs via a clone abundance weighting mechanism, significantly improving classification performance, particularly in the sensitivity of early cancer detection.

These methods have demonstrated promising results across various stages, yet there remains room for further improvement. To further enhance the performance of early cancer detection, we propose a multi-instance deep neural network model named AutoTFCNNY. This model combines the strengths of Transformer and CNN, balancing the extraction of global and local features from TCR sequences. In AutoTFCNNY, the Transformer plays a central role by leveraging its powerful global feature extraction capabilities to effectively model the dependencies between distant positions within TCR sequences. Specifically, the encoder component [36] employs multi-head self-attention, enabling the model to focus more on the most relevant TCR sequences without excluding or ignoring other TCR sequences. Instead, it models the interactions among them to understand the collective effects of multiple TCR sequences or the entire TCR repertoire. It is noteworthy that Transformers typically rely on relatively larger training datasets. When the training data size is insufficient or the feature noise is high, Transformers may exhibit insufficient generalization when distinguishing local key regions due to the lack of inherent inductive biases present in some cognitive neural networks [37]. Therefore, when the data size is limited and local patterns are crucial for classification tasks, Transformers may have certain limitations in capturing local detailed features. These local detailed features may be essential for distinguishing cancer-associated TCRs from normal TCRs. To address this limitation, AutoTFCNNY incorporates a CNN architecture. CNN utilizes a local receptive field strategy, scanning along the sequence dimension with convolution kernels to effectively capture subtle yet biologically meaningful local features. This is attributed to the inherent inductive bias of CNNs towards local patterns, enabling the model to stably capture discriminative local information even in environments with limited data or high noise levels [38–40]. Experimental results demonstrate that the combination of Transformer and CNN provides good performance for AutoTFCNNY. The integration enhances the model’s ability to recognize cancer-related features, significantly improving detection accuracy and robustness. Additionally, it also enables the model to perform effectively in terms of noise adaptability and generalization capability.

Leveraging this optimized architecture, we achieved the highest overall AUC scores on most datasets for cancer detection tasks. The main contributions of this study are summarized as follows:

- We propose a novel multi-instance deep neural network that combines Transformer and CNN architectures, enabling both global and local feature extraction from TCR sequences, and significantly improving the accuracy, sensitivity, and generalizability of cancer detection.
- AutoTFCNNY outperforms most baseline methods for TCR sequence-based cancer detection and achieves the highest AUC scores across various types of cancer datasets.
- In external validation on five independent cancer test sets, AutoTFCNNY demonstrates excellent generalization ability and consistently outperforms other comparative models.

The remainder of this paper is organized as follows. “Materials and methods” presents the details of our datasets and methodological framework. “Model” describes the model architecture, parameter settings, and implementation environment. “Results” reports the experimental results and evaluates the model’s applicability and generalizability on multiple independent datasets. Finally, “Discussion” and “Conclusion” provide a discussion and summary of the work.

## Materials and methods

### Dataset information

The cancer data sets used in this study were obtained from the immuneACCESS online database (IA) of Adaptive Biotechnologies, which is a public TCR-seq data repository. IA is a genomics online database that focuses on storing and sharing immune receptor and immunome data. The database integrates data from multiple research laboratories and projects, including information on the immune systems of humans and other species. In addition, our healthy control sample data set was derived from the study of Xu et al. [32]. These data can be downloaded from the following link: https://github.com/Bioinformatics7181/DeepLION/tree/master/Data/THCA/TrainingData. The above-mentioned dataset covers 22 types of cancer and a group of healthy control samples. For more information, see Table 1. (see S1 File for more details).

**Table 1:** Information about the dataset.

Types of Cancer	Sample Size	Data Type	Data Source
BC	114	TCR-seq	IA
BL	187	TCR-seq	IA
BRCA	220	TCR-seq	IA
CC	128	TCR-seq	IA
CRC	120	TCR-seq	IA
DLBCL	92	TCR-seq	IA
GBM	48	TCR-seq	IA
HCC	56	TCR-seq	IA
KS	472	TCR-seq	IA
Lung	111	TCR-seq	IA
Lung ADC	158	TCR-seq	IA
MCC	72	TCR-seq	IA
MF	96	TCR-seq	IA
Melanoma	281	TCR-seq	IA
NSCLC	224	TCR-seq	IA
OC	230	TCR-seq	IA
OS	41	TCR-seq	IA
PCa	94	TCR-seq	IA
PDAC	16	TCR-seq	IA
RCC	53	TCR-seq	IA
SCLC	52	TCR-seq	IA
UBC	117	TCR-seq	IA
Health	214	TCR-seq	Xu et al. [32]

*BC: Brain Cancer; BL: Burkitt Lymphoma; BRCA: Breast Cancer; CC: Cervical Cancer; CRC: Colorectal Cancer; DLBCL: Lymphoid Neoplasm Diffuse Large B-cell Lymphoma; GBM: Glioblastoma Multiforme; HCC: Hepatocellular Carcinoma; KS: Kaposi sarcoma; Lung: Lung Cancer; Lung ADC: Lung Adenocarcinomas; MCC: Merkel Cell Carcinoma; MF: Mycosis fungoides; Melanoma: Melanoma; NSCLC: Non-small Cell Lung Cancer; OC: Ovarian Cancer; OS: Osteosarcoma; PCa: Prostate Cancer; PDAC: Pancreatic Ductal Adenocarcinoma; RCC: Renal Cell Carcinoma; SCLC: Small-cell Lung Cancer; UBC: Urothelial Bladder Cancer; TCR-seq: T Cell Receptor-sequencing; IA: immuneACCESS online database.

### Data preprocessing

In order to effectively extract features from TCR sequence data and improve the overall performance of the model, the following steps were performed during the data preprocessing stage [29]:

1. **Length filter**: Eliminates sequences that are too short (less than 10) or too long (more than 24);
2. **Special character processing**: Sequences containing special characters (e.g., X, +, *, etc.) may affect data quality due to technical or sequencing errors, so such sequences are removed to improve the cleanliness and consistency of the dataset;
3. **Follow IMGT nomenclature**: According to the ImMunoGeneTics (IMGT) standard [41], delete incomplete sequences that do not begin with cysteine (C) or end with phenylalanine (F) to maintain the consistency and integrity of the sequence;
4. **TCR sequence screening**: The top 100 high-frequency TCR sequences in each sample were selected. Because high-frequency TCR sequences may have important biological activity in immune responses [42];
5. **Sample screening**: After completing the above steps, remove samples with fewer than 10 TCR sequences. Ensure that each sample has a sufficient number of sequences.

### Multi-instance learning

As a branch of machine learning, Multi-Instance Learning (MIL) was first proposed by Dietterich et al. [43] in the study of drug activity prediction. In recent years, this method has been widely applied to the detection of cancer-related TCRs [30–34]. The standard assumption of MIL is that a bag is labeled as positive if there is at least one positive instance in the bag; otherwise, the bag is labeled as negative. Predicting whether each TCR repertoire is associated with cancer can be described as a MIL problem. In our MIL framework, each bag corresponds to a subject’s TCR repertoire, encompassing all TCR sequences experimentally obtained from that patient. This TCR repertoire is treated as a collective sample set, representing the characteristics of the patient’s immune system. Each TCR sequence corresponds to an instance, which is the basic component of a bag and stands for a specific TCR sequence. Different instances in a bag represent various characteristics of the patient’s immune system. The bag label is used to indicate whether the entire TCR repertoire is associated with a particular type of cancer. Bag labels take values in {0,1}—e.g., 0 denotes non-cancer (negative bag), and 1 denotes cancer (positive bag). After preprocessing, each data sample’s bag label is assigned as 0 or 1 according to whether it originates from a healthy cohort or a cancer cohort, respectively. The collection of bag samples is represented as:

$$\left\{({𝐗}_{1},Y_{1}),({𝐗}_{2},Y_{2}),\ldots ,({𝐗}_{n},Y_{n})\right\}$$ (1)

where each ${𝐗}_{i}$ represents a collection of bag instances, and *Y*_*i*_ is the corresponding label. The determination of *Y*_*i*_ is based on the instances in ${𝐗}_{i}$. The core objective of MIL is to learn a linear classifier *f* that can accurately predict *Y*_*i*_ based on ${𝐗}_{i}$. The classifier *f* is defined as follows:

$$\tilde{Y}=P\left(Y=1|\left\{{𝐌}_{1},\ldots ,{𝐌}_{k}\right\}\right)=\sigma^{\prime }\left({𝐖}^{f^{T}}{\left[{\tilde{y}}_{1},\ldots ,{\tilde{y}}_{k}\right]}^{T}+b^{f}\right)$$ (2)

where ${𝐌}_{k}$ denote the *k*-th TCR feature matrix, and $P\left(Y=1|\left\{{𝐌}_{1},\ldots ,{𝐌}_{k}\right\}\right)$ represents the probability of being cancer-associated. $\sigma^{\prime }(x)$ is the sigmoid function, ${\tilde{y}}_{k}$ represents the score of the *k*-th TCR instance, and $\left[{\tilde{y}}_{1},\ldots ,{\tilde{y}}_{k}\right]$ is a vector containing the scores of all instances in the bag. The parameters ${𝐖}^{f}\in {\mathbb{R}}^{k}$ and $b^{f}\in \mathbb{R}$ are the weight matrix and bias of *f*, respectively.

The probability obtained through the sigmoid function reflects the association between the bag and the target cancer. When $\tilde{Y}$ is greater than 0.5, the model classifies the sample as cancer-associated; otherwise, it classifies it as non-cancer.

### TCR coding

To effectively encode TCR sequences into numerical matrices containing antigen-binding specificity, especially of CDR3, is key to accurately recognizing cancer-related TCRs. To accurately capture the biochemical characteristics of biomolecules, we used a coding method based on principal component analysis (PCA). Specifically, Beshnova et al. [29] used PCA to generate a 20×15 feature matrix from 531 amino acid indices [44], characterizing the biochemical properties of amino acids. Considering that the Beshnova matrix was derived from the largest number of amino acid indices, it contains the most biochemical information (explaining over 95% of the variance in the data), and it has been validated in methods such as DeepCAT [29], DeepLION [32], and DeepLION2 [34], exhibiting excellent performance. Therefore, in this study, we employed the Beshnova matrix (dimension *d* = 15) to encode amino acids and vectorize the input sequences for further processing (see S1 File).

## Model

### Transformer-based encoder and CNN model

**Fig 1:**
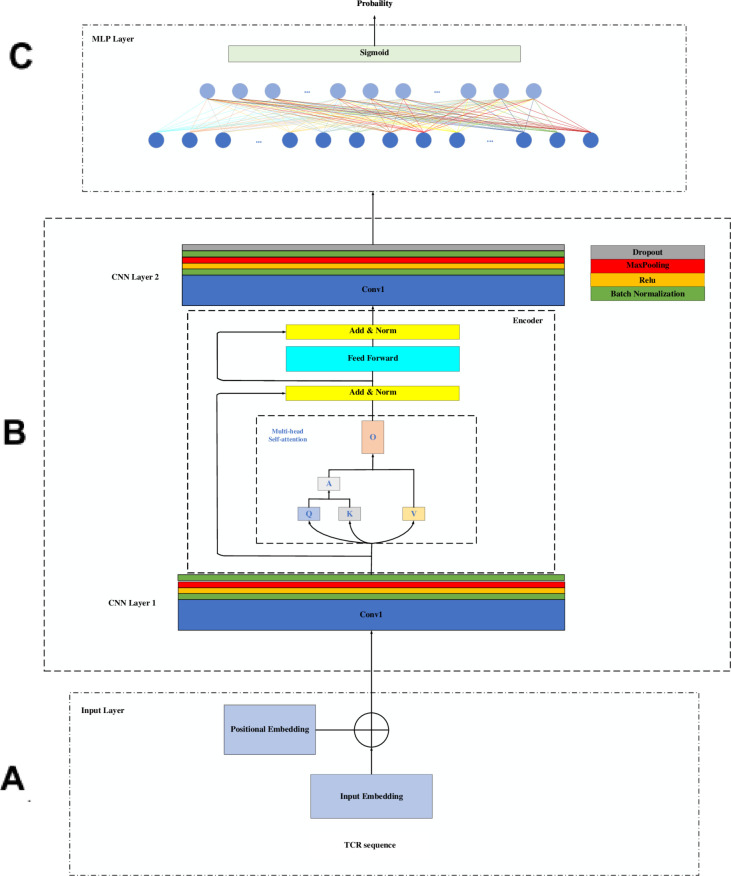
AutoTFCNNY framework diagram. (A) **Data encoding and position embedding.** The original TCR sequence is embedded as a 24 × 15 matrix after PCA encoding, and the sequence position information is incorporated into the representation of each amino acid through position encoding to enhance the model’s ability to process sequential data. (B) **TCR feature extraction.** Local features are extracted using a two-layer CNN, and global dependencies in the TCR sequence are modeled using a Transformer encoder. Local and global features are combined to improve prediction capabilities. (C) **MLP module.** On the output after feature extraction, the MLP module performs classification through a fully connected layer and outputs a probability value for cancer prediction using a sigmoid activation function to output the final prediction result.

Specifically, as shown in Fig 1, AutoTFCNNY consists of an embedding layer, a position encoding layer, a CNN layer, a Transformer Encoder layer, and an MLP module:

**Embedding layer.** The embedding layer is defined as $𝐄\in {\mathbb{R}}^{d\times k}$, where *d* = 24 and *k* = 15 represent the sequence length and embedding dimension, respectively. Each amino acid is mapped to a 15-dimensional vector, and these vectors are subsequently arranged into a 24×15 tensor to meet the input requirements of the subsequent processing layers. The embeddings are randomly initialized using a uniform distribution, and padding is applied to maintain a constant sequence length.

**Position encoding layer.** Position encoding is employed to incorporate positional information into the input sequence, which is essential for processing sequence data. The position encoding matrix **PE** is defined as [36]:

$${PE}_{\left(pos,2i\right)}=\sin\left(\frac{pos}{10000^{2i/d_{\text{model}}}}\right)$$ (3)

$${PE}_{\left(pos,2i+1\right)}=\cos\left(\frac{pos}{10000^{2i/d_{\text{model}}}}\right)$$ (4)

where *pos* denotes the position within the sequence, *i* represents the dimension index, and $d_{\text{model}}$ specifies the embedding dimension. To reduce the risk of overfitting, a Dropout ratio of 0.3 is applied. The maximum allowable sequence length for position encoding is set to 100.

**Convolutional layer.** The convolutional layer serves as the core component for feature extraction and is defined as follows:


$${𝐲}^{\left(m\right)}(j)=\sum_{n=1}^{C_{\text{in}}}\sum_{i=0}^{k-1}{𝐖}^{\left(m,n\right)}(i)\cdot {𝐱}^{\left(n\right)}(j+i)+{𝐛}^{\left(m\right)}$$

where ${𝐲}^{\left(m\right)}(j)$ represents the value of the *m*-th output feature map at position *j*, $C_{\text{in}}$ denotes the number of input channels, ${𝐖}^{\left(m,n\right)}(i)$ denotes the weight of the convolution kernel connecting the *n*-th input channel to the *m*-th output channel at position *i*, ${𝐱}^{\left(n\right)}$ denotes the *n*-th input feature map, *k* denotes the size of the convolution kernel (*k* = 8), and ${𝐛}^{\left(m\right)}$ denotes the bias term for the *m*-th output channel.

Following the convolution operation, batch normalization is applied:


$$\text{BN}(𝐱)=\gamma \left(\frac{𝐱-\mu }{\sqrt{\sigma^{2}+\epsilon }}\right)+\beta$$

where μ and $\sigma^{2}$ are the within-batch sample mean and variance, respectively, γ and β are learnable parameters, and ϵ is a small constant added to prevent division by zero.

Subsequently, the Rectified Linear Unit (ReLU) activation function is applied:


ReLU(𝐱)=max(0,𝐱)

An adaptive max pooling layer is utilized to reduce the spatial dimension to a fixed size of 1, effectively performing global max pooling:


$${𝐲}^{\left(m\right)}=\max_{0\leq i<S}{𝐱}^{\left(m\right)}(i)$$

where ${𝐲}^{\left(m\right)}$ is the pooled output for the *m*-th feature map, ${𝐱}^{\left(m\right)}$ is the input to the pooling operation, and *S* is the spatial dimension of the input feature map.

The weights of the convolutional and fully connected layers are initialized using the Xavier initialization method [45]:


$${𝐰}_{i}^{\left(l\right)}\sim 𝒩\left(0,\frac{2}{M_{l-1}+M_{l}}\right)$$

where ${𝐰}_{i}^{\left(l\right)}$ represents the weight parameter of the *l*-th layer, and *M*_*l*_ is the number of neurons in the *l*-th layer.

**Transformer encoder layer.** The core of the encoder is the self-attention mechanism, which allows the model to dynamically attend to important information across different positions in the sequence. Multi-head attention extends this mechanism by enabling the model to simultaneously capture information from multiple representation subspaces. For each attention head *i* (i=1,…,h), the input 𝐗 is linearly transformed into a query (**Q**), key (**K**), and value (**V**)representation [30]:

$${𝐐}_{i}=𝐗{𝐖}_{i}^{q},\;{𝐊}_{i}=𝐗{𝐖}_{i}^{k},\;{𝐕}_{i}=𝐗{𝐖}_{i}^{v},\;i=1,\ldots ,h$$ (5)

where ${𝐖}_{i}^{q}$, ${𝐖}_{i}^{k}$, and ${𝐖}_{i}^{v}$ are the learnable weight matrices for the *i*-th head.

The scaled dot-product attention is then computed by measuring the similarity between the query ${𝐐}_{i}$ and all the keys ${𝐊}_{i}$, followed by the softmax operation to produce attention weights:

$$Attention({𝐐}_{i},{𝐊}_{i},{𝐕}_{i})=softmax\left(\frac{{𝐐}_{i}{𝐊}_{i}^{T}}{\sqrt{d_{k}}}\right){𝐕}_{i}$$ (6)

where *d*_*k*_ is the dimension of the key vector, introduced to stabilize the gradient.

The outputs of all attention heads are concatenated and passed through a final linear transformation:

$$MultiHead(𝐐,𝐊,𝐕)=Concat\left({head}_{1},\ldots ,{head}_{h}\right){𝐖}^{O}$$ (7)

where each attention head is defined as:

$${head}_{i}=Attention({𝐐}_{i},{𝐊}_{i},{𝐕}_{i})$$ (8)

and ${𝐖}^{O}$ is the weight matrix of the output linear transformation.

Only a single encoder layer is used , with a hidden layer size of 30, 10 attention heads (see S1 File).

**MLP module.** After feature extraction by the preceding layers, the final feature representation is obtained as $𝐱\in {\mathbb{R}}^{hidden\_size}$, which serves as the input to the MLP module. The MLP module consists of one fully connected layer that maps the processed features to the output space. Specifically, the feature 𝐱 is linearly transformed using the weight matrix $𝐖\in {\mathbb{R}}^{1\times hidden\_size}$ and the bias b∈ℝ:

z=𝐖𝐱+b (9)

where z is the linearly transformed output.

To prevent overfitting, a Dropout layer is applied prior to the MLP module, which randomly drops a portion of neurons during training. The dropout rate is here set to 0.4 (S1 File). The output *z* is then passed through a logistic activation function (sigmoid):

$$\sigma (z)=\frac{1}{1+e^{-z}}$$ (10)

The sigmoid function maps *z* to the range [0,1], resulting in a probability value related to cancer.

### Other settings

**Binary cross-entropy with logits loss (BCEWithLogitsLoss):** The Binary Cross-Entropy with Logits Loss is defined as:

$$BCEWithLogitsLoss(z,y)=\max(z,0)-z\cdot y+\ln\left(1+e^{-|z|}\right)$$ (11)

where *z* is the raw output value (logits) before activation, and *y* is the actual label or target value, taking values of 0 or 1 for binary classification problems. The unified decision threshold is set to 0.5, such that if the predicted probability exceeds 0.5, the sample is classified as cancer-associated; otherwise, it is classified as non-cancer.

**Adam optimizer:** The model is optimized using the Adam optimizer, which combines an adaptive learning rate and momentum-based approach [46]. The update rule is given as:

$$\left\{ \begin{array}{c} m_{t}=\beta_{1}m_{t-1}+(1-\beta_{1})g_{t}\\ v_{t}=\beta_{2}v_{t-1}+(1-\beta_{2})g_{t}⊙g_{t}\\ \theta_{t}=\theta_{t-1}-\alpha \frac{m_{t}/(1-\beta_{1}^{t})}{\sqrt{v_{t}/(1-\beta_{2}^{t})}+\varepsilon } \end{array}\right.$$ (12)

where *g*_*t*_ is the gradient at time step *t*, *m*_*t*_ and $v_{t}$ represent the exponential moving averages of the first- and second-order moments of the gradient *g*_*t*_, respectively. Here, $\beta_{1}$ and $\beta_{2}$ are the smoothing coefficients with default values $\beta_{1}=0.9$ and $\beta_{2}=0.999$. $\theta_{t}$ denotes the model parameter at time step *t*, and *α* is the learning rate, set to α=0.0005. The term $\varepsilon =10^{-8}$ is introduced to prevent division by zero.

**Training configuration:** The total number of training epochs is set to 2000, with a batch size of 64.

**Early-stopping mechanism:** To prevent overfitting, an early-stopping mechanism is implemented with a patience value of 300. This means that if the validation set performance does not improve for 300 consecutive epochs, training is terminated early, and the model with the best performance is saved for testing.

### Metrics

To comprehensively evaluate the performance of the proposed model, the following key evaluation metrics are adopted: Accuracy (ACC), Sensitivity (SEN), Specificity (SPE), and the Area Under the ROC Curve (AUC). The mathematical definitions of ACC, SEN, and SPE are as follows:

$$ACC=\frac{TP+TN}{TP+FP+TN+FN}$$ (13)

$$SEN=\frac{TP}{TP+FN}$$ (14)

$$SPE=\frac{TN}{TN+FP}$$ (15)

where TP, TN, FP, and FN represent the number of true positive cases, true negative cases, false positive cases, and false negative cases, respectively.

In addition, the model performance is further evaluated using the Area Under the Receiver Operating Characteristic (ROC) Curve (AUC), which reflects the model’s ability to distinguish between positive and negative samples under varying thresholds.

Classification model performance is comprehensively evaluated using the AUC value under the ROC curve, which reflects the model’s ability to distinguish between positive and negative cases under various thresholds; the closer the value of AUC is to 1, the better the model performs in categorizing positive and negative cases.

To ensure an accurate evaluation of the model, we employ an iterated *K*-fold cross-validation procedure with data shuffling. The process is detailed as follows: the *K*-fold cross-validation is repeated *n* times. Before each iteration of the *K*-fold validation, the data are randomly shuffled and divided into *K* equal parts. In each iteration, *K* − 1 parts are used as the training set, while the remaining part serves as the test set. The training set is further split into training and validation subsets in an 8:2 ratio. These subsets are subsequently used as the final training data to optimize the model. The overall model performance is quantified as the average of the evaluation metrics over *n* iterations:

$$\overset{―}{metric}=\frac{1}{n}\sum_{i=1}^{n}{metric}_{i}$$ (16)

where *n* = 100 and *K* = 5. This iterative approach ensures robust and reliable model assessment metrics.

### Configuration information

We implemented the proposed method using Python 3.8, PyTorch 2.5.1, and CUDA 12.4 within a deep learning framework. The computing system was equipped with an AMD Ryzen 9 7900X 12-Core Processor CPU, 64 GB RAM, and an NVIDIA GeForce RTX 4090 GPU with 24 GB of memory. The operating system used was Ubuntu 22.04.3 LTS.

## Results

### Comparison model selection

To evaluate the performance of AutoTFCNNY, we compared it with several state-of-the-art methods, including iCanTCR [35], DeepLION [32], MINN-SA [33], DeepLION2 [34], TransMIL [47], and BiFormer [48] (as summarized in Table 2).

**Table 2:** Summary of comparison models.

Model	TCR Prediction?	Incorporates Attention Mechanism?	Sparsity of Instance Distribution?	Instance Correlation?
AutoTFCNNY	Yes	Yes	No	Yes
iCanTCR	Yes	No	No	Yes
DeepLION	Yes	No	No	No
DeepLION2	Yes	Yes	Yes	Yes
MINN-SA	Yes	Yes	Yes	No
TransMIL	No	Yes	No	Yes
BiFormer	No	Yes	Yes	Yes

DeepLION, DeepLION2, and MINN-SA are embedded-space MIL methods specifically designed for TCR prediction. DeepLION processes variable-length TCR data through alternating convolutional filters and 1-max pooling operations, and it has been shown to outperform earlier caTCR prediction methods such as DeepCAT [29]. MINN-SA addresses the sparsity of caTCRs by leveraging the sparsemax function to selectively focus on key TCRs while ignoring less relevant ones. This method has demonstrated superior performance compared to popular MIL approaches for this task. DeepLION2 further improves upon DeepLION by introducing content-based sparse self-attention to select the top-*k* most relevant TCRs, effectively modeling instance correlations. In addition, a contrastive learning strategy is incorporated to refine the attention matrix and prevent the model from focusing on non-cancer-associated TCRs. In contrast to the above approaches, iCanTCR adopts a deep learning framework that combines CNN and LSTM architectures to capture both spatial and sequential features within TCR sequences. Furthermore, it incorporates an abundance-weighting strategy to highlight the importance of high-abundance TCRs in cancer detection, significantly improving classification performance, particularly in early cancer detection. TransMIL and BiFormer, on the other hand, were not originally designed for TCR prediction tasks. TransMIL is a Transformer-based MIL model initially developed for whole-slide image (WSI) classification. It integrates the self-attention mechanism into its MIL framework to effectively capture correlations among instances and has demonstrated impressive performance in WSI classification. BiFormer, a more recent content-based sparse attention method proposed in the computer vision domain, introduces a bi-level routing attention mechanism that significantly improves classification accuracy compared to other self-attention-based approaches.

To ensure a fair comparison with these models, we employed preprocessing strategies similar to those used in the DeepLION2 study. (as described in the DeepLION2 paper [34] and its GitHub). Specifically, we made appropriate adjustments to ensure compatibility with the evaluation dataset while retaining each model’s core architecture and parameters as much as possible. To evaluate the performance of AutoTFCNNY and the comparison models, we employed commonly used metrics from machine learning and statistical analysis, including accuracy (**ACC**), sensitivity (**SEN**), specificity (**SPE**) and the Area Under the ROC Curve (**AUC**). Given the relatively small sample size of the datasets used in our experiments, we further validated the model’s performance through 5-fold cross- validation.

### Model performance on different types of cancer datasets

**Fig 2:**
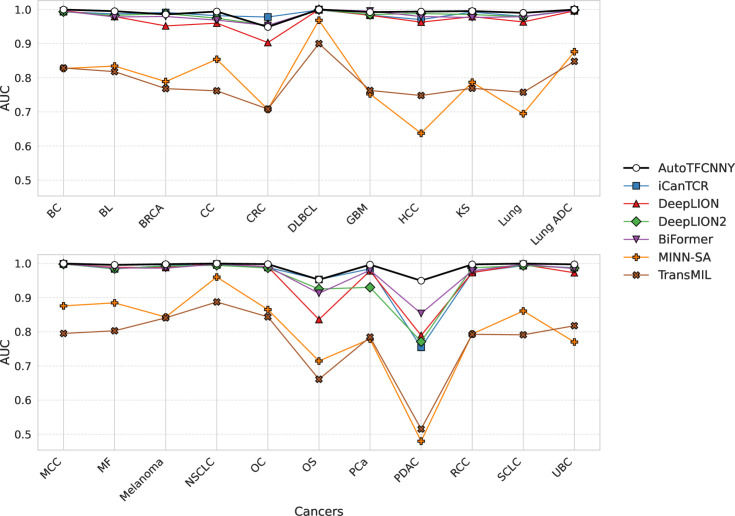
Comparison of all models on different cancer types. This plot shows the distribution of the average AUC values across 22 cancer datasets, providing a global view of the performance of each model. Each point represents the average AUC value of the model on this cancer across 100 rounds of 5-fold cross-validation.

**Fig 3:**
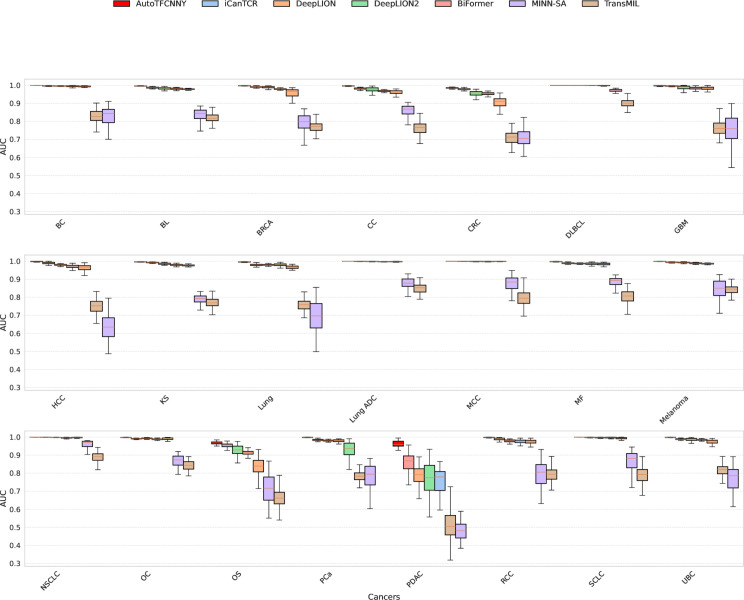
Boxplots of AUC values for different models on a dataset of 22 cancer types. The boxplots compare the variability of model performance across different cancer types. Different colors are used to indicate model type, and within each group of cancers, they are sorted by median AUC.

To ensure the reliability and applicability of the results, we performed 100 rounds of 5-fold cross-validation experiments on the model and evaluated the performance of each model on 22 cancer datasets. All the results of the experiments are shown in Table 3 (see S1 File for detailed results), while Fig 2 and 3 illustrate the average AUC values for each model across different cancer types, as well as their respective distributions.

**Table 3:** Average performance of the models on the 22 cancer datasets.

Types of Cancers	iCanTCR	DeepLION	DeepLION2	MINN-SA	TransMIL	BiFormer	AutoTFCNNY
BC	0.993433	0.996690	0.993467	0.826799	0.828767	0.995431	**0.999710**
BL	0.986909	0.979262	0.982598	0.833838	0.818026	0.978120	**0.994948**
BRCA	**0.991234**	0.951857	0.988165	0.788794	0.768063	0.979484	0.985651
CC	0.981747	0.959941	0.974397	0.853912	0.761838	0.968807	**0.994024**
CRC	**0.977921**	0.903100	0.953512	0.707248	0.708677	0.953220	0.948664
DLBCL	0.998698	0.999846	0.999416	0.968530	0.900772	0.999956	**1.000000**
GBM	0.983398	0.982869	0.985333	0.751443	0.761747	**0.995438**	0.992300
HCC	0.970411	0.962926	0.988019	0.636972	0.748404	0.979302	**0.993783**
KS	0.992607	0.978910	0.985140	0.787147	0.769145	0.976441	**0.995308**
Lung	0.979515	0.963606	0.978812	0.695223	0.757057	0.978922	**0.990132**
Lung ADC	0.998883	0.995809	0.998151	0.875815	0.849089	0.997332	**0.999621**
MCC	0.998709	0.998168	0.998124	0.875616	0.795586	0.999173	**0.999952**
MF	0.984308	0.988909	0.985080	0.884574	0.802808	0.987092	**0.995937**
Melanoma	0.993631	0.988294	0.992386	0.842883	0.841306	0.986392	**0.998096**
NSCLC	0.995488	0.999117	0.994628	0.960191	0.887424	0.998303	**0.999929**
OC	0.988358	0.990771	0.987100	0.865436	0.843796	0.990887	**0.998194**
OS	**0.953319**	0.835986	0.925323	0.715094	0.661120	0.912717	0.952545
PCa	0.984323	0.978568	0.930232	0.778543	0.784345	0.980372	**0.996686**
PDAC	0.754460	0.789355	0.771043	0.479940	0.514154	0.853645	**0.949650**
RCC	0.974588	0.973557	0.987377	0.793510	0.792349	0.978774	**0.997364**
SCLC	0.993839	0.996756	0.994047	0.860526	0.791513	0.997918	**0.999933**
UBC	0.987800	0.972939	0.986713	0.769264	0.818024	0.985136	**0.997517**

* The maximum AUC values for the evaluation metrics in the comparison models are shown in bold. AUC: Area Under the ROC Curve.

From the experimental results, AutoTFCNNY shows good performance, with an average AUC value higher than other models for 18 of the 22 cancers. In addition, the average AUC value on the 22 cancer type datasets is higher than 0.94, and the AUC value for 18 of the cancer types is even higher than 0.99 (Fig 2, Fig 3, Table 3 and S1 File). Specifically, AutoTFCNNY performed particularly well on the DLBCL dataset, with an average AUC of 1.000 and high robustness (mean standard deviation of 0) (see S1 File). And on the PDAC dataset, AutoTFCNNY still outperformed other models (see Table 3), despite the high imbalance and feature sparsity of this dataset (see Table 1 and S1 File).

In contrast, the iCanTCR, DeepLION, DeepLION2, and BiFormer models also achieved strong results, ranking among the top performers in this comparison and demonstrating notable advantages on specific cancer datasets. Among them, iCanTCR outperformed other models in BRCA, CRC and OS, especially in CRC (see Fig 2, Fig 3, Table 3 and S1 File). Specifically, the AUC value on the CRC dataset reached 0.9961, significantly outperforming all other models. This can be attributed to its abundance weighting strategy, highlighting its advantage in handling complex data. DeepLION processes variable-length TCR sequences using convolutional filters and pooling layers, and also achieves good results on most cancer datasets, but is significantly lower on a few cancer datasets (such as CRC and OS, PDAC) (see Fig 2, Fig 3 and Table 3). Interestingly, compared with DeepLION, the improved version DeepLION2 outperforms DeepLION on most data sets, especially CRC and OS (see Fig 2, Fig 3 and Table 3), which indicates that its improved content-based sparse self-attention mechanism and contrastive learning strategy are effective in these cancer types and have better adaptability to these cancer types. However, it is worth noting that it fails to show the same adaptability on some cancer datasets (such as PCA and PDAC) (see Fig 2, Fig 3 and Table 3), and its performance is lower than that of DeepLION and other models. This may be because sparse modeling relies too heavily on instances that are highly correlated with the target and fails to capture the interactions among a few instances. Meanwhile, an overemphasis on non-key features, although it performed well on some datasets, is susceptible to noise on datasets with feature sparsity or small sample sizes due to the bias that the contrastive learning strategy may introduce for some non-cancer-related TCRs. Although BiFormer is not specifically designed for the characteristics of TCR data, it achieves a good balance between classification accuracy and computational efficiency through a two-level routing attention mechanism, and performs well on most cancer types, especially on certain cancer types (such as GBM), outperforming other models (see Fig 2, Fig 3 and Table 3).

It is worth noting that the performance of each model is generally limited on the OS and PDAC datasets (see Fig 2, Fig 3 and Table 3). Although AutoTFCNNY still maintains its leading position on the PDAC dataset, its AUC value (0.9497) is lower compared to other cancer types. The reasons for this phenomenon include: insufficient sample size. The OS and PDAC datasets have small sample sizes (see Table 1), which causes the model to tend to predict the majority class (healthy samples) and ignore the minority class (cancer samples), thereby reducing sensitivity. Insufficient feature extraction. The TCR sequences in these datasets may lack significant cancer-specific features, limiting the model’s feature extraction ability.

In addition, some models (e.g., MINN-SA and TransMIL) performed poorly in terms of AUC on multiple datasets (Fig 2, Fig 3 and Table 3). This is mainly because MINN-SA fails to adequately model the correlation between TCR sequences. Although its sparse attention mechanism helps suppress noise, it may be insufficient in scenarios with complex instance interactions. The self-attention mechanism of TransMIL was originally designed to process whole slide pathology image data, while TCR sequence data lacks significant spatial information, which leads to its insufficient adaptability on TCR datasets and limits its performance to a certain extent.

**Fig 4:**
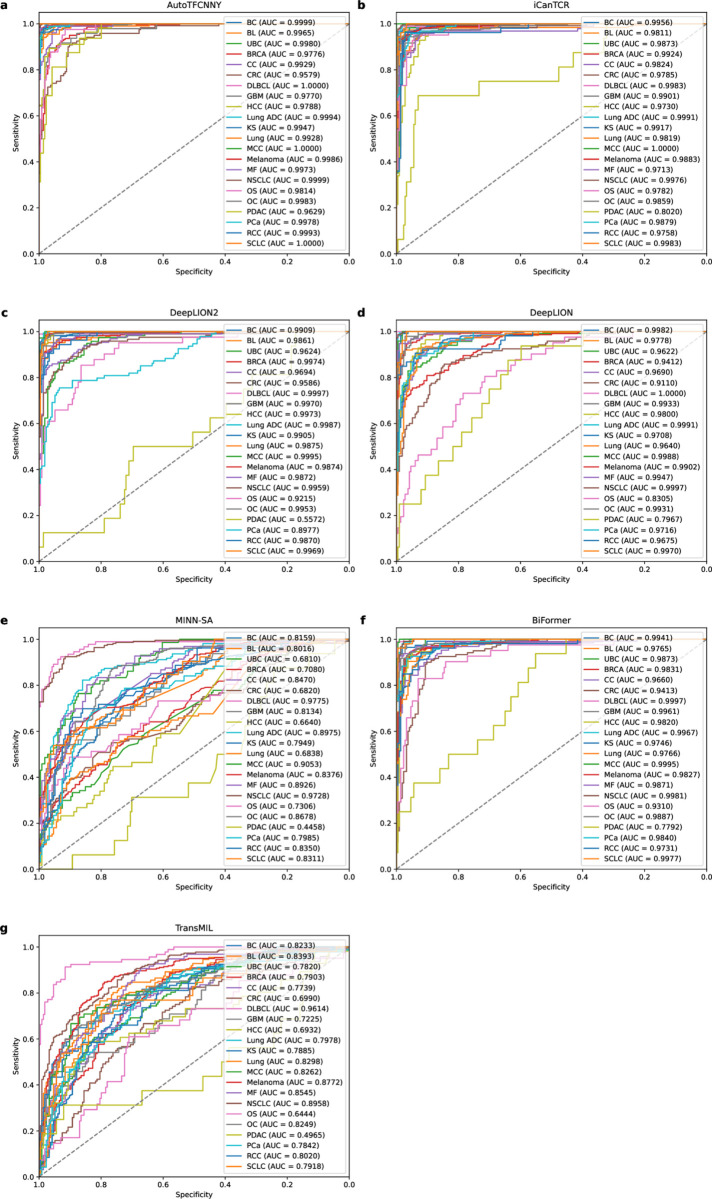
ROC curves of the model on 22 cancer datasets. This figure shows the ROC curves of AutoTFCNNY and other models on 22 cancer datasets in 5-fold cross-validation experiments. Each subplot shows the ROC curve of a different model on a specific dataset.

The AUC box plot in Fig 4 further reveals the stability and advantages of AutoTFCNNY. It performs slightly better than other comparison models on most cancer datasets, especially on the PDAC dataset. It is worth noting that although AutoTFCNNY has higher AUC values than other models on most cancer datasets, other models also show competitiveness on some cancer datasets.

### Performance of AutoTFCNNY model on DeepLION dataset

To thoroughly ensure the fairness of the experiment and the generalisation performance of the model, we also conducted a comparative experiment on the data sets published on DeepLION’s GitHub). These data sets mainly come from the TCR-seq data of the clinical database of Geneplus Technology Ltd. in Shenzhen, and include two sets of cancer sample data sets and one set of healthy sample data sets (see Table 4 for details). Furthermore, these data sets are divided into training sets, validation sets and test sets. The final test results are shown in Table 5.

**Table 4:** DeepLION dataset information.

Types of Cancer	Sample Size	Cell Type
Lung	184	PBMCs and TILs
THCA	170	PBMCs and TILs
Health	260	PBMCs

* Lung: Lung Cancer; THCA: Thyroid Cancer; PBMCs: Peripheral Blood Mononuclear Cells; TILs: Tumor-Infiltrating Lymphocytes.

**Table 5:** Performance of AutoTFCNNY and DeepLION on the DeepLION dataset.

Cancer	Model	ACC	SEN	SPE	AUC
Lung	AutoTFCNNY	**0.8864**	**0.8378**	**0.9216**	**0.9534**
	DeepLION	0.8068	0.8108	0.8039	0.8887
THCA	AutoTFCNNY	**0.8953**	**0.8250**	0.9565	**0.9717**
	DeepLION	0.8837	0.8000	0.9565	0.9560

* The maximum value of the evaluation metric in the comparison model is displayed in bold. ACC, accuracy; SEN, sensitivity; SPE, specificity; AUC, area under the receiver operating characteristic curve.

**Fig 5:**
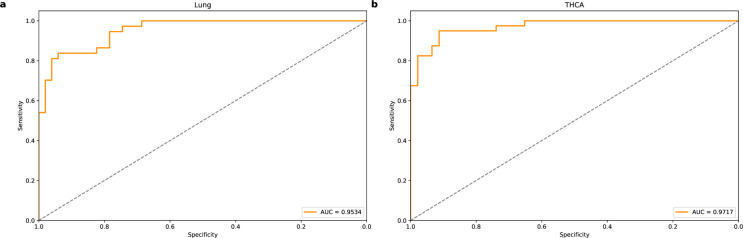
ROC curve for AutoTFCNNY. The ROC curves of the AutoTFCNNY model on the Lung and THCA datasets show the model’s classification performance on these two datasets. (a) The ROC curve of the AutoTFCNNY model on the Lung dataset, with an AUC value of 0.9534. (b) The ROC curve of the AutoTFCNNY model on the THCA dataset, with an AUC value of 0.9717.

**Fig 6:**
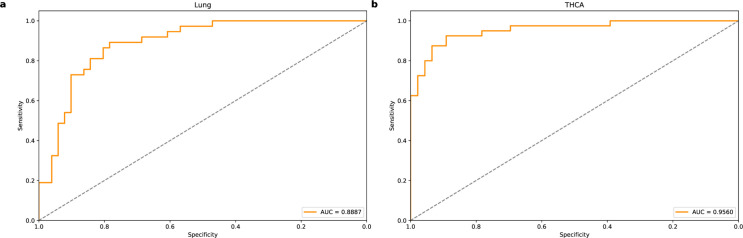
ROC curve for DeepLION. The ROC curves of the DeepLION model on the Lung and THCA datasets reflect the model’s performance on these two datasets. (a) The ROC curve of the DeepLION model on the Lung dataset has an AUC value of 0.8887. (b) The ROC curve of the DeepLION model on the THCA dataset has an AUC value of 0.9560.

Table 5 shows the performance of the two models on the Lung and THCA cancer datasets. Figs 5 and 6 show the ROC curves of AutoTFCNNY and Deeplion for the two cancers, respectively. The AutoTFCNNY model shows better overall performance. In particular, on Lung, the AUC value reached 0.9534, and the values of accuracy, sensitivity, and specificity were 0.8864, 0.8378, and 0.9216, respectively, all of which exceeded those of DeepLION. On THCA, the two models performed equally well, and both maintained a high level of specificity, reaching 0.9565. In summary, AutoTFCNNY outperforms DeepLION on the DeepLION dataset, especially on the Lung dataset, where AutoTFCNNY shows more prominent performance. Although the performance of the two models is similar in some datasets (such as THCA), AutoTFCNNY’s advantage in terms of accuracy and sensitivity makes it perform better.

### Model testing

To comprehensively validate the generalization performance of AutoTFCNNY, we supplemented the existing training set with additional cancer sample data collected from other published studies, along with 51 new healthy control samples, to construct an independent test dataset. This section will explore the dataset composition, the model’s performance on different test datasets, and threshold selection and performance evaluation. We collected five types of cancer data—UBC, BRCA, NSCLC, Melanoma, and CRC—from the immune sequencing database (immuneACCESS) (details provided in Table 6, with further information available in S1 File). Additionally, 51 healthy samples were obtained from DeepLION to serve as the healthy control group in the new test dataset.

**Table 6:** Test data set information.

Types of Cancers	Sample Size	Data Type	Data Source
UBC	12	TCR-seq	IA
BRCA	23	TCR-seq	IA
NSCLC	66	TCR-seq	IA
Melanoma	21	TCR-seq	IA
CRC	10	TCR-seq	IA
Health	51	TCR-seq	DeepLION

BRCA: Breast Cancer; CRC: Colorectal Cancer; Melanoma: Melanoma; NSCLC: Non-small Cell Lung Cancer; UBC: Urothelial Bladder Cancer; TCR-seq: T Cell Receptor-sequencing; IA: immuneACCESS online database.

During the training process, we constructed the training set using previously curated cancer and healthy samples (as shown in Table 1) and trained the AutoTFCNNY model, ultimately saving the best-performing model for subsequent testing. Concurrently, to comprehensively validate the model’s performance and compare the strengths and weaknesses of different approaches, we selected several comparative models that demonstrated outstanding performance during training—including iCanTCR, BiFormer, and DeepLION2. All models were trained on the same training set to ensure the comparability and fairness of the evaluation results. Subsequently, we assessed the detection performance of each model on a newly constructed independent test dataset, with the decision threshold uniformly set at 0.5. Table 7 provides a detailed presentation of the performance of each model on the new test dataset.

**Table 7:** Performance of Models on the New Dataset (Threshold = 0.5).

Types of Cancers	Model	ACC	SEN	SPE	AUC
UBC	AutoTFCNNY	**0.9167**	**0.8333**	**1.0000**	**0.9931**
	iCanTCR	0.7083	0.4167	**1.0000**	0.9861
	BiFormer	0.8333	0.7500	0.9167	0.9583
	DeepLION2	0.8333	0.6667	**1.0000**	0.9722
BRCA	AutoTFCNNY	0.7174	0.4348	**1.0000**	**1.0000**
	iCanTCR	0.8261	0.6522	**1.0000**	0.9662
	BiFormer	**0.8478**	**0.6957**	**1.0000**	0.9924
	DeepLION2	0.7609	0.5217	**1.0000**	0.9905
NSCLC	AutoTFCNNY	0.7009	0.4697	**1.0000**	**0.9923**
	iCanTCR	0.6581	0.3939	**1.0000**	0.9587
	BiFormer	**0.7179**	**0.5152**	0.9804	0.9623
	DeepLION2	0.5726	0.2424	**1.0000**	0.9201
Melanoma	AutoTFCNNY	**1.0000**	**1.0000**	**1.0000**	**1.0000**
	iCanTCR	0.9762	0.9524	**1.0000**	**1.0000**
	BiFormer	0.9762	0.9524	**1.0000**	**1.0000**
	DeepLION2	0.6190	0.3333	0.9048	0.8617
CRC	AutoTFCNNY	**0.9000**	**0.8000**	**1.0000**	**0.9800**
	iCanTCR	0.6000	0.2000	**1.0000**	0.6000
	BiFormer	0.8500	0.7000	**1.0000**	0.8500
	DeepLION2	0.6000	0.2000	**1.0000**	0.8500

^*^ ACC: Accuracy; SEN: Sensitivity; SPE: Specificity; AUC: Area Under the ROC Curve. Maximum values of evaluation metrics within each cancer type are highlighted in bold.

As can be observed from Table 7, the detection performance of each model varies across different cancer types. Overall, AutoTFCNNY demonstrates exceptionally high SPE and AUC values across all datasets, indicating its superior global discriminative ability to effectively distinguish between healthy and cancerous samples. Specifically, in the UBC, Melanoma, and CRC datasets, AutoTFCNNY not only achieves advantages in ACC and SEN but also maintains near-perfect AUC values, suggesting its more precise and robust capture of relevant features. In the BRCA and NSCLC datasets, BiFormer performs better in ACC and SEN, while AutoTFCNNY excels in SPE and AUC. Notably, on these two datasets, all models exhibit relatively poor performance in ACC and SEN but perform exceptionally well in SPE and AUC metrics. This indicates that all models almost flawlessly identify healthy samples, while their sensitivity in detecting cancerous samples is relatively insufficient. Potential reasons for this phenomenon include limited sample size, dataset imbalance, and the inherent biological heterogeneity of cancer. Additionally, on the Melanoma dataset, iCanTCR, BiFormer, and AutoTFCNNY all perform exceptionally well, with AutoTFCNNY achieving a “perfect” performance with all metrics equal to 1. However, this may also reflect the risk of overfitting due to the small sample size or highly aligned training features. Nevertheless, it is important to note that, despite AutoTFCNNY’s impressive performance in the aforementioned test results, achieving certain metrics of 1 is highly uncommon in real-world applications, particularly in the field of medical diagnosis, where variability and uncertainty are prevalent. Therefore, such performance may indicate that the test set is small or not representative, or that the model may have encountered overfitting during training. This implies that the model might have overly learned the features of the training data without acquiring the generalization capability needed to perform well on unseen samples.

**Fig 7:**
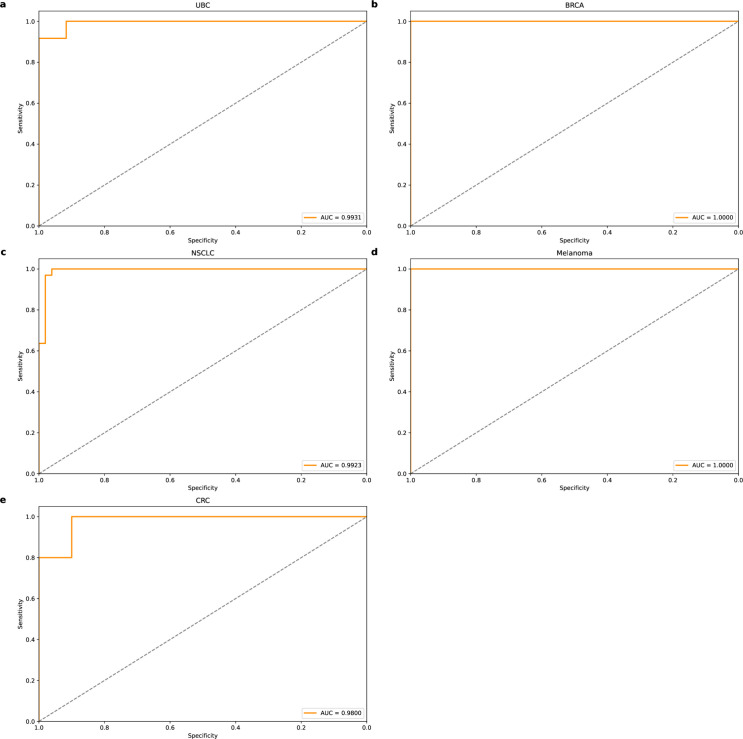
ROC curve of AutoTFCNNY on a new test dataset. ROC curves of AutoTFCNNY on multiple independent test datasets, further verifying the generalisation ability and stability of the model. (a) ROC curve of the AutoTFCNNY model on the UBC dataset, with an AUC of 0.9931. (b) ROC curve of the AutoTFCNNY model on the BRCA dataset, with an AUC of 1. (c) ROC curve of the AutoTFCNNY model on the NSCLC dataset with an AUC of 0.9923. (d) ROC curve of the AutoTFCNNY model on the Melanoma dataset, with an AUC of 1. (e) ROC curve of the AutoTFCNNY model on the CRC dataset, with an AUC of 0.98.

Fig 7 shows the ROC curve of AutoTFCNNY with a threshold of 0.5 for the new dataset. Overall, AutoTFCNNY performed well in this test. In particular, the AUCs for UBC, NSCLC, and CRC detection were all greater than or equal to 0.98. It is worth noting that the AUCs for BRCA and Melanoma detection both reached a perfect 1. Such results are extremely rare in practical applications. Such results are uncommon in practical applications. Therefore, despite the significant outcomes, we must remain cautious that the features learned by the model may not adequately encompass or represent all biological characteristics associated with cancer.

The above test analysis is based on a fixed threshold of 0.5, and it may not be optimal to use a fixed threshold of 0.5 to determine the results in actual testing. Different types of cancer and different clinical scenarios may require different threshold settings, because cancer detection usually involves a trade-off between false positives and false negatives. A high threshold may reduce the false positive rate (i.e., reduce the number of cases of cancer that are misdiagnosed), but at the same time may lead to an increase in the false negative rate (i.e., a missed diagnosis of an actual cancer). Conversely, a low threshold may reduce false negatives, but may lead to an increase in false positives, which increases the psychological and financial burden on patients.

In order to more accurately apply it to cancer detection, we use the Combined Score, which is the simple average of accuracy, sensitivity and specificity, to determine the optimal threshold for model performance:

$$\text{Combined Score}=\frac{\text{Accuracy}+\text{Sensitivity}+\text{Specificity}}{3}$$ (17)

The optimal threshold range is determined by calculating the overall score for each threshold:

$$\left[\theta_{\min},\theta_{\max}]=\arg\max_{\theta \in \Theta }\text{Combined Score}(\theta \right)$$ (18)

**Fig 8:**
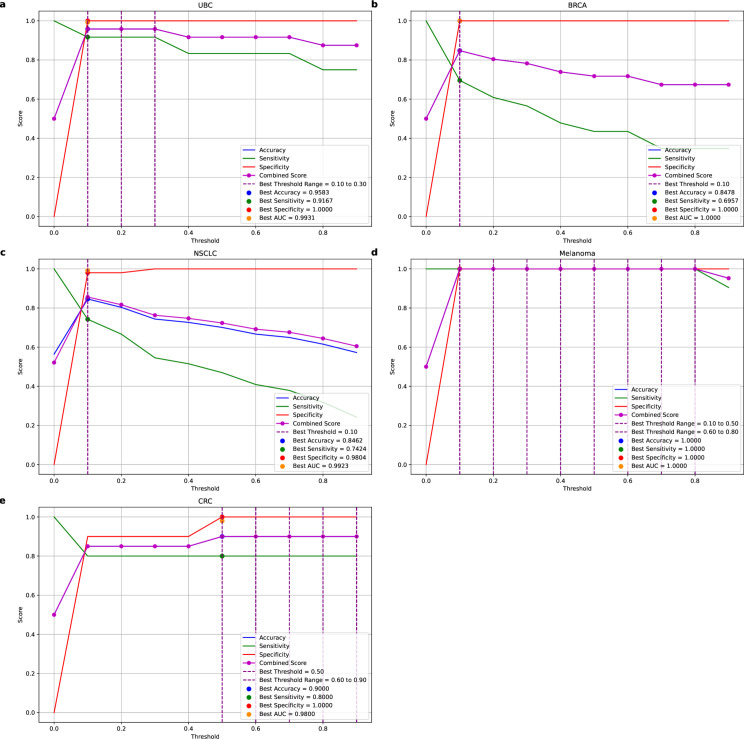
Performance metrics of AutoTFCNNY as a function of threshold. This figure shows AutoTFCNNY’s performance metrics on a new test dataset at different thresholds. The solid purple line shows the trend of the combined score under different thresholds. The purple dotted line indicates the optimal threshold of the AutoTFCNNY model, at which the accuracy, sensitivity and specificity of the model remain at a high level. Accuracy is represented by the solid blue line. Sensitivity is represented by the solid red line. Specificity is represented by the solid green line. (a) Performance metrics of the AutoTFCNNY model at different thresholds on the UBC dataset. (b) Performance metrics of the AutoTFCNNY model on the BRCA dataset at different thresholds. (c) Performance metrics of the AutoTFCNNY model on the NSCLC dataset at different thresholds. (d) Performance metrics of the AutoTFCNNY model on the Melanoma dataset at different thresholds. (e) Performance metrics of the AutoTFCNNY model on the CRC dataset at different thresholds.

The performance indicators of each model were plotted against the threshold value (as shown in Fig 8).

The Fig 8 illustrates the trends of ACC , SEN, SPE , and the combined score across varying thresholds for AutoTFCNNY. At the threshold indicated by the purple dashed line, where the combined score reaches its peak, the model achieves a well-balanced trade-off between accuracy, sensitivity, and specificity. For example, on the UBC dataset, when the threshold is set between 0.1 and 0.3, the model maintains high ACC (0.9583), high SEN (0.9167), and perfect SPE (1.0000), achieving better overall performance compared to the default threshold of 0.5.

On the BRCA and NSCLC datasets, sensitivity fluctuates significantly with the threshold, while specificity remains consistently high. For cancer screening applications, if the clinical goal is to reduce missed diagnoses, the threshold can be lowered to increase sensitivity, albeit with an acceptance of higher false positive rates. On the Melanoma and CRC datasets, due to the model’s excellent performance, the choice of threshold has a relatively small impact on the performance metrics. However, the potential risk of overfitting remains, requiring further validation on larger, more diverse external datasets.

Overall, AutoTFCNNY demonstrates outstanding performance across multiple independent test datasets, particularly achieving near-perfect detection on the Melanoma dataset. The model also maintains high accuracy and sensitivity on the UBC and CRC datasets. However, on the BRCA and NSCLC datasets, the model exhibits relatively lower sensitivity but achieves high specificity and AUC, indicating its strong ability to accurately identify non-cancer cases. Depending on clinical needs, if reducing missed diagnoses (improving sensitivity) is prioritized, adjusting the threshold or employing weighted loss functions can be considered. Conversely, to minimize misdiagnoses, specificity can be maintained at high levels while supplementing sensitivity through additional diagnostic methods.

Moreover, for results showing “perfect” performance on small datasets or datasets with features highly similar to the training set, further validation is needed on larger and more heterogeneous datasets to eliminate potential overfitting or biases due to dataset limitations. Future work should explore additional cancer types and more comprehensive clinical data, while continuing to address issues of threshold selection and data imbalance. This will ensure that the AutoTFCNNY model attains greater reliability and scalability in real-world clinical applications.

## Discussion

In this study, we developed a detection model named AutoTFCNNY based on a multi-instance learning approach. Structurally, the AutoTFCNNY model integrates a Transformer encoder with a CNN, aiming to enhance detection accuracy by leveraging the Transformer’s global perception capabilities alongside the CNN’s local feature extraction abilities.

Although AutoTFCNNY performed well in the experiments, there are still some limitations in our research. Due to experimental conditions and resource limitations, only a small number of cancer patient sample data were used in the detection experiments in this study, which greatly limited the breadth of the test and the verification of the model’s generalization ability. In addition, the data used were all from public literature, which may only represent the characteristics of a specific population and are not generalizable. These characteristics may include other characteristics in addition to cancer-related characteristics. To this end, we also found a small number of cancer samples and healthy control group sample data from other published literature to construct an independent test set to further verify the performance of the model. The results show that AutoTFCNNY achieved good results in the four indicators of Accuracy, Sensitivity, Specificity, and AUC. Although these supplementary experiments verified the preliminary effectiveness of the model, it is not difficult to see that the model trained with a small number of samples is not sufficient to cover all the biomarker characteristics of a certain cancer. Therefore, we still have reason to doubt whether the research results based on a small number of samples are representative of all cancer patients. Although AutoTFCNNY performed well in the preliminary test, the limitations of a small number of samples may have prevented the model from fully capturing the broader and more complex cancer characteristics and biomarkers. In this case, the generalization ability of AutoTFCNNY and its application in different populations still need to be further verified. Secondly, in terms of data processing, it is difficult to rule out the influence of other potential causes of the sample. Therefore, we aim to provide methods and ideas, and verify the feasibility of the model concept we propose. Ideally, we hope to collect more diverse cancer data by expanding the sample size and self-sequencing, which will not only further verify the reliability and generality of the model, but also help to improve the accuracy and practicality of AutoTFCNNY in actual clinical applications. Future research should also consider using large datasets with multiple factors and multiple information to enhance the applicability and accuracy of the model.

**Fig 9:**
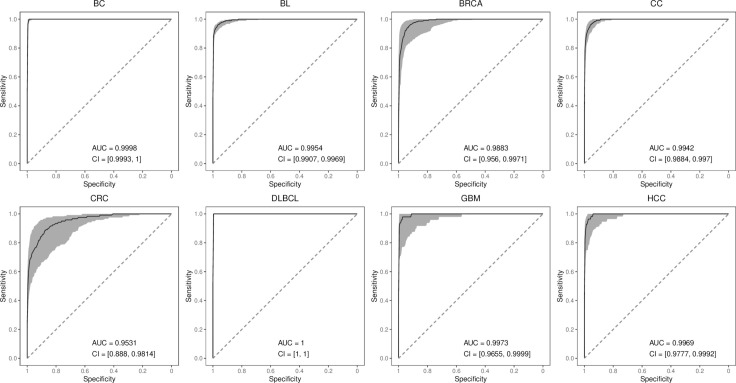
ROC curves for AutoTFCNNY model, superimposed with confidence intervals with coefficient 0.95.

The experimental results show that AutoTFCNNY has good performance in the detection of 22 types of cancer. To comprehensively evaluate the robustness of AutoTFCNNY, we performed 100 rounds of 5-fold cross-validation experiments for each of the 22 different types of cancer, and obtained the ROC curve and the confidence interval with a coefficient of 0.95 for each cancer. The following is a superimposed plot of the ROC curve and the confidence interval with a coefficient of 0.95 for some cancers (see Fig 9). The ROC curve and the confidence interval with a coefficient of 0.95 for the remaining cancers can be found in S1 File.

As shown in Fig 9, AutoTFCNNY generally exhibits good robustness (see S1 File). This is especially evident in the detection of BC, BL, and DLBCL, where AutoTFCNNY has the smallest fluctuations and the narrowest confidence intervals, indicating high robustness. It performs moderately well in the detection of BRCA, HCC, and CC. It performed poorly in CRC and GBM, with AutoTFCNNY showing large fluctuations in its results and wide confidence intervals (CIs), indicating large performance fluctuations and low robustness of the model in different cross-validation rounds.

**Fig 10:**
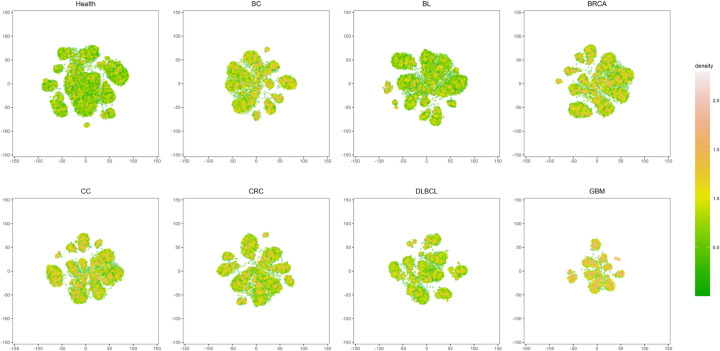
Sample-specific difference heat map. The light yellow indicates a higher density value, representing a high-density region of TCR sequences. In these regions, there are more TCR sequence points clustered, which may indicate an increase in the diversity of TCR sequences in the cancer state. Green indicates a lower density value, showing a lower density of TCR sequences. There are fewer TCR sequence points in these regions, which may reflect the stability of TCR sequences in this state.

To further analyze the reasons for the differences in the performance of the model on different cancer datasets, we plotted the specific difference heat maps of 22 cancer groups and healthy controls according to the visualization method proposed by Yokota et al. [49] These heat maps reflect the differences in the characteristic distribution and density changes of TCR sequences in different groups after t-SNE dimensionality reduction. The following are some of the specific difference heat maps of cancer samples (see Fig 10), and the remaining cancer sample specific difference heat maps are shown in S1 File.

Overall, the heat maps of TCR sequence distribution for different cancers show significant diversity, with each sample having multiple small regions of high density that differ from the healthy control group. There are also differences in the distribution characteristics between different types of cancer. Each cancer has its own specific TCR sequence distribution pattern, which may be related to the immune characteristics of different cancers (see S1 File).

Specifically, the heat map of the control group shows a wide distribution area, with an overall green color, indicating that the density value is low in most areas. Although there are a few yellowish areas, they are more small clusters, and the overall distribution is slightly scattered. This indicates that the TCR sequences in the two-dimensional space are scattered, with no obvious high-density clusters, reflecting the stability of TCR sequences in a healthy state. The DLBCL distribution area is larger than the other cancers, with a yellowish overall color and a few green areas, showing multiple small areas of high density, and the clustering characteristics of the TCR sequence are obvious. Compared with the healthy control group, there are obvious characteristic differences. AutoTFCNNY can accurately extract key TCR feature information and has excellent performance (the average AUC value is 1, the highest among the 22 cancers, and it is highly robust, see S1 File and Fig 9). Although some information in the high-dimensional space may be lost during the dimensionality reduction process when using t-SNE, AutoTFCNNY can still effectively distinguish the healthy group from the DLBCL group. This shows that AutoTFCNNY has strong nonlinear expression ability and can capture complex patterns and relationships, which enables AutoTFCNNY to capture subtle differences that t-SNE cannot show when dealing with high-dimensional features. The distribution areas of the four cancer types BC, BL, BRCA, and CC are similar, with an overall yellowish color and a small number of green areas. The density values of most areas are high, indicating that their TCR sequences are highly aggregated in the two-dimensional spatial aggregation area. Compared with the healthy control group, there are obvious characteristic differences. The model can effectively extract some key TCR feature information and perform well (the average AUC value is greater than 0.98, see S1 File). It is worth noting that CRC has a similar distribution to the four cancers mentioned above, and there are also visible differences from the healthy control group. However, compared to the other four cancers, the model performed poorly in the detection of CRC (the average AUC value was only 0.94, see S1 File), and did not effectively learn the characteristics associated with CRC. The reason for this is that, on the one hand, there may be large heterogeneity within CRC samples, making it difficult for the model to extract consistent TCR features. On the other hand, CRC may have more complex biological characteristics, making it difficult to accurately distinguish between them based solely on TCR sequence information. This also explains why AutoTFCNNY is not suitable for all cancers. Although some distributional similarities can be observed, more in-depth research is needed to identify more representative and specific features in related cancers. The GBM sample size was the smallest, with only 4800 TCR sequences (see Table 1). The heat map distribution area was small, but the overall color was light yellow, which was the brightest color compared to other cancers. This means that the TCR sequence density is highly concentrated. Compared with the healthy control group, the feature differences are significant. The model can effectively extract TCR features specific to GBM and perform well (with an average AUC value of 0.99, see S1 File). This indicates that AutoTFCNNY can effectively extract key features and accurately identify GBM even with limited sample data. However, in order to improve the robustness of the model and ensure diagnostic accuracy, it is necessary to increase the sample size, which will help improve the model’s ability to learn specific features and improve its stability on a wider sample.

Of course, it is undeniable that these 22 cancers are themselves a very complex type of cancer. Their pathogenesis involves the interaction of multiple genetic, immune, and environmental factors, which makes prediction more challenging. Due to their more complex etiology, this may lead to greater difficulty for the model in processing the data. In addition, feature engineering may also play a role in performance degradation. More complex and refined feature engineering may be required in cancer detection to capture cancer-related TCR-specific features and information. If the feature engineering is insufficient or the features are not selected properly, the model may not be able to fully utilize the available information, which may affect performance.

In future research, we can improve the predictive performance of these cancers in the following ways. First, we can collect more samples of cancer data sets to balance the data sets. We can achieve this through multi-center cooperation, data sharing, and clinical trials. Second, we can study the pathogenesis of these cancers in greater depth and carefully design and select features that better reflect their characteristics. Finally, we can try to use more advanced machine learning algorithms and explore the introduction of more complex model architectures or integrated methods that combine multiple models to better handle complexity and data imbalance, thereby improving prediction performance. At the same time, parameter adjustment and cross-validation can be performed to optimize the performance of the model. By taking these measures, we will hopefully further improve the prediction accuracy and application scope of the model, and improve the accuracy of cancer diagnosis.

## Conclusion

In the context of effectively detecting early-stage cancers and leveraging deep learning algorithms, this study introduces a multi-instance deep neural network model named AutoTFCNNY, which is based on Transformer and CNN architectures. The paper provides a comprehensive introduction and analysis of the data sources, data processing methods, research methodologies, neural network architecture of the model, selection of key parameters, performance evaluation, and testing assessment. Experimental results demonstrate that AutoTFCNNY exhibits excellent performance in detecting 22 types of cancers. However, it is evident that for certain cancers with limited sample sizes in the dataset, the model’s detection performance is less pronounced, which, in a way, underscores the model’s efficiency. Additionally, through the analysis of heatmaps highlighting the characteristic differences among various sample groups, we conducted a more in-depth exploration of the dataset. Finally, we actively discussed the issues and limitations encountered during the study and proposed several solutions. These efforts are expected to provide effective tools and methodologies for the advancement of early cancer diagnosis, treatment, and vaccine design.

## Supporting information

S1 File Supplementary materials. This single PDF file contains S1–S6 Figs and S1–S3 Tables, including: ROC curve of AutoTFCNNY on 22 cancers with 95% confidence intervals, sample characteristic difference heat map, ablation experiments, and summary of datasets.
(PDF)
