# Supplementary material for: AutoTFCNNY: A multi-instance neural network for enhanced early cancer detection using TCR data
Source: PLoS One. 2025 Oct 8;20(10):e0326253. doi: 10.1371/journal.pone.0326253 (PMC12507200; doi:10.1371/journal.pone.0326253)
Supplement: S1 File — This single PDF file contains S1–S6 Figs and S1–S3 Tables, including: ROC curve of AutoTFCNNY on 22 cancers with 95% confidence intervals, sample characteristic difference heat map, ablation experiments, and summary of datasets. (PDF) [file pone.0326253.s001.pdf]

PLOS ONE

Supplementary Materials for

AutoTFCNNY: A Multi-Instance Neural Network for Enhanced Early Cancer Detection Using TCR Data

DonghongYang[1], XinPeng[1], Yiming Zhou[2], ShenglanPeng[1*]
1 Jingdezhen Ceramic University, Jingdezhen, China
2 Base and Byte Biotechnology Company Ltd, Beijing, China
*Corresponding author. Email: *solfix123@163.com

The PDF file includes:

Fig. S1. ROC curve of AutoTFCNNY on 22 cancers, with confidence interval for the coefficient of 0.95.
Fig. S2. Sample characteristic difference heat map.
Fig. S3. Ablation experiment – number of PCA.
Fig. S4. Ablation experiment – Dropout.
Fig. S5. Ablation experiment– number of attentions.
Fig. S6. Ablation experiment– number of encoders.
Table S1. Average performance of the models on the 22 cancer datasets.
Table S2. Summary information of experimental dataset.
Table S3. Summary information of test datasets.

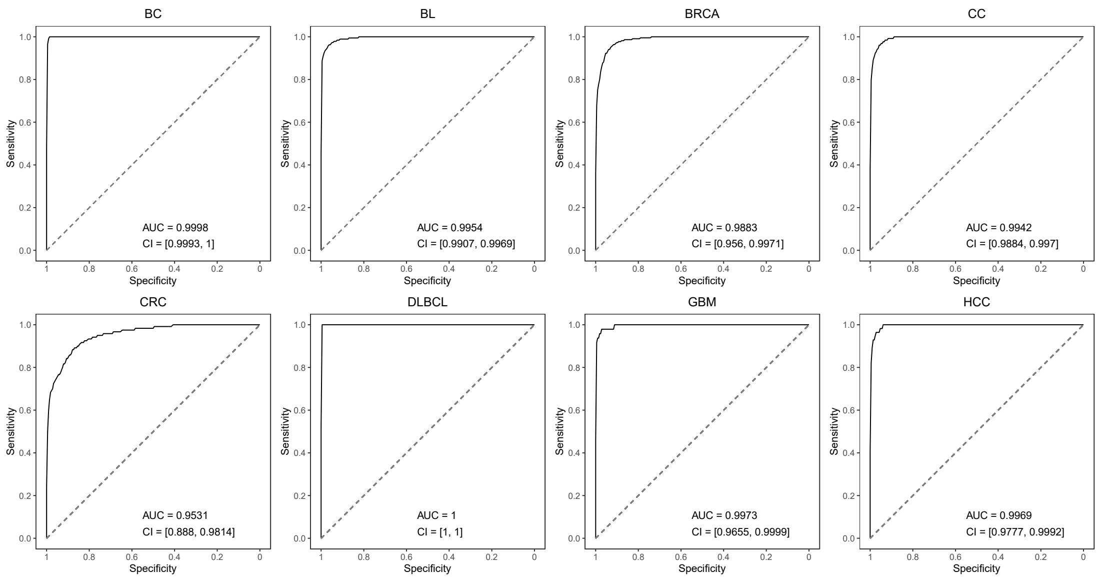
BC
AUC = 0.9998
CI = [0.9993, 1]
BL
AUC = 0.9954
CI = [0.9907, 0.9969]
BRCA
AUC = 0.9883
CI = [0.956, 0.9971]
CC
AUC = 0.9942
CI = [0.9884, 0.997]
CRC
AUC = 0.9531
CI = [0.888, 0.9814]
DLBCL
AUC = 1
CI = [1, 1]
GBM
AUC = 0.9973
CI = [0.9655, 0.9999]
HCC
AUC = 0.9969
CI = [0.9777, 0.9992]
Sensitivity
Specificity

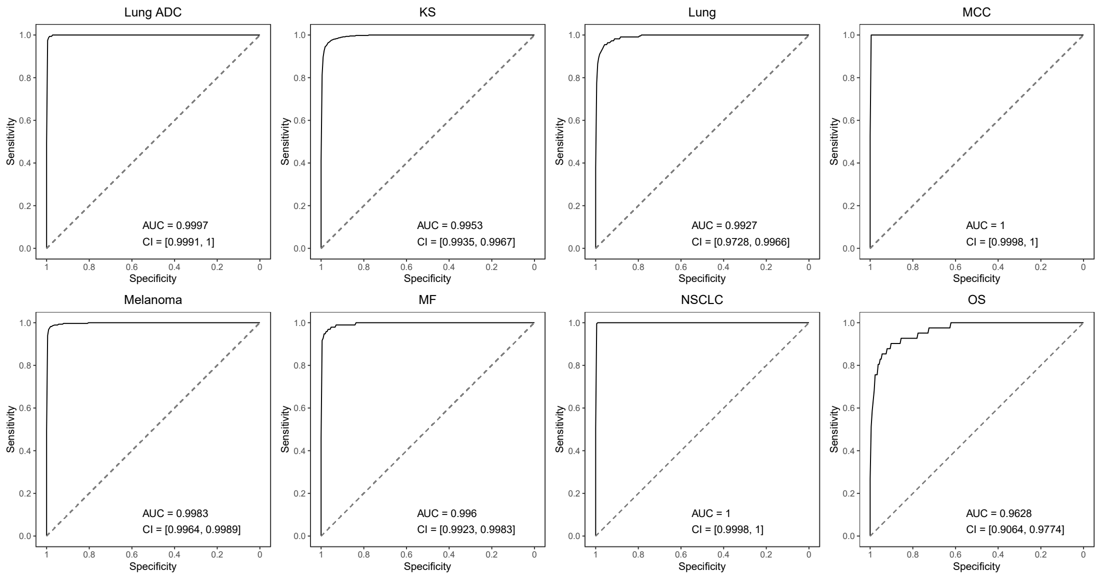
Lung ADC
AUC = 0.9997
CI = [0.9991, 1]
KS
AUC = 0.9953
CI = [0.9935, 0.9967]
Lung
AUC = 0.9927
CI = [0.9728, 0.9966]
MCC
AUC = 1
CI = [0.9998, 1]
Melanoma
AUC = 0.9983
CI = [0.9964, 0.9989]
MF
AUC = 0.996
CI = [0.9923, 0.9983]
NSCLC
AUC = 1
CI = [0.9998, 1]
OS
AUC = 0.9628
CI = [0.9064, 0.9774]
Sensitivity
Specificity

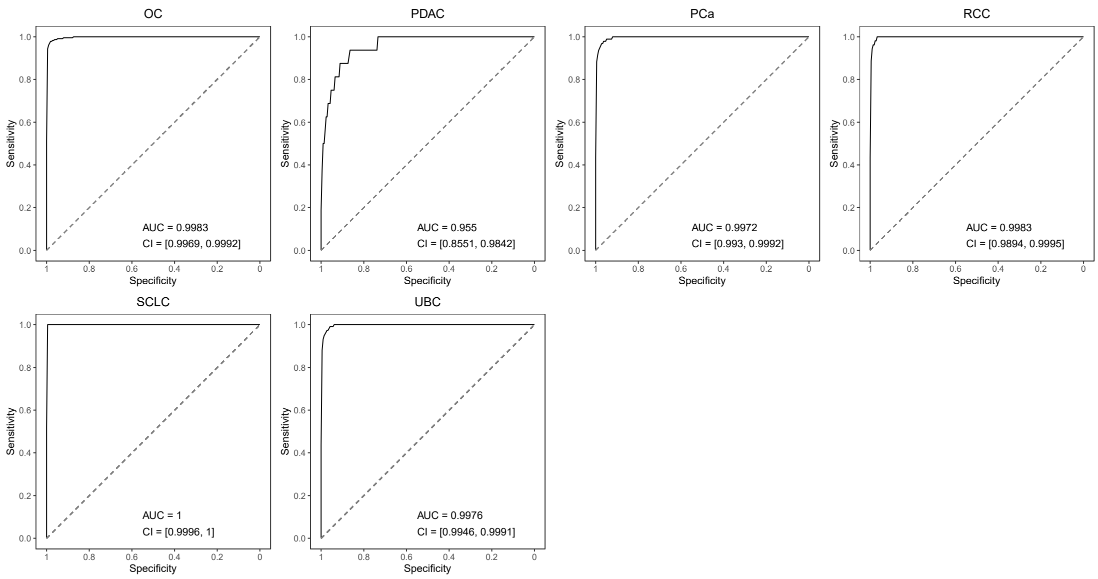

Fig. S1. ROC curve of AutoTFCNNY on 22 cancers, with confidence interval for the coefficient of 0.95.

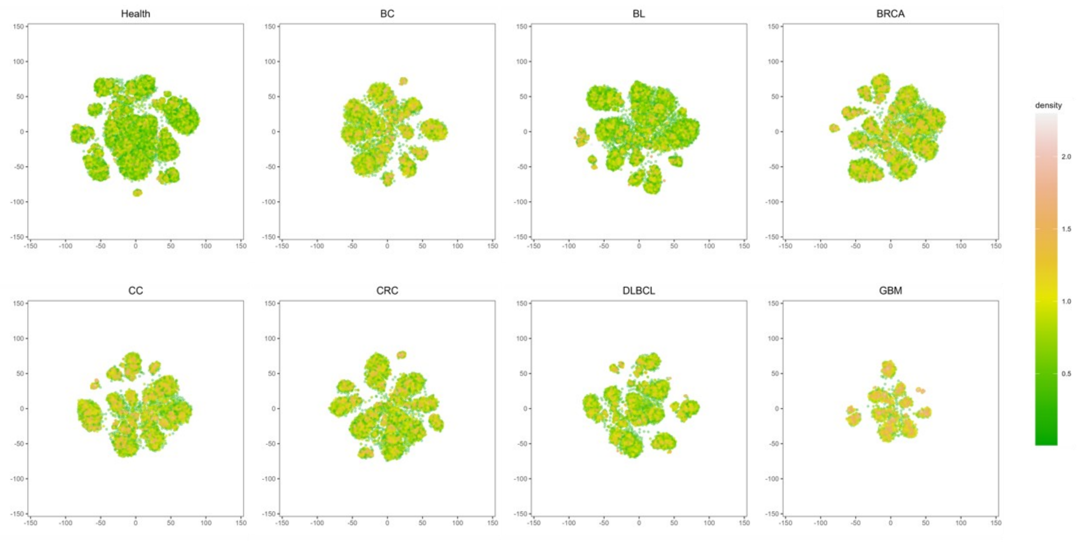
Health
BC
BL
BRCA
CC
CRC
DLBCL
GBM
density
2.0
1.5
1.0
0.5

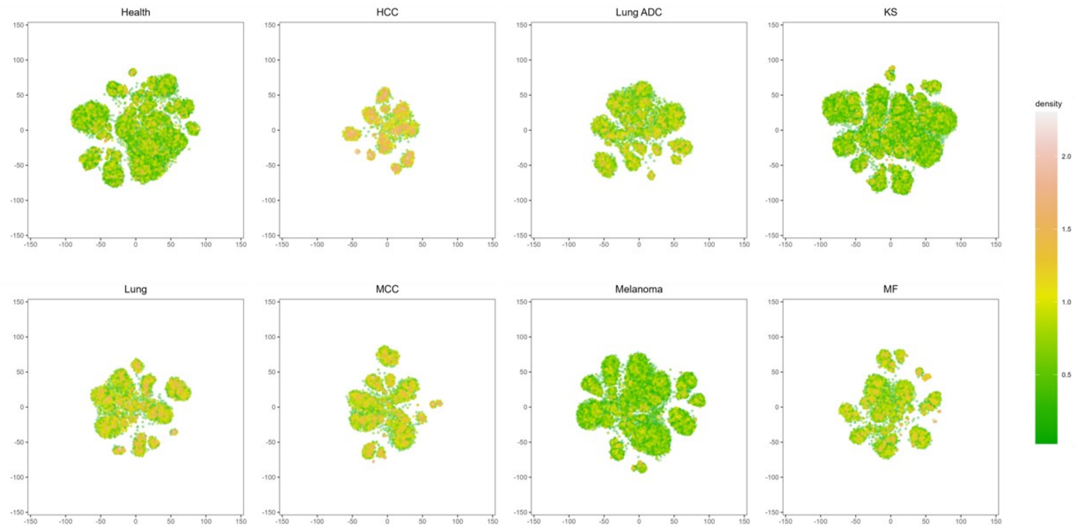
Health
HCC
Lung ADC
KS
Lung
MCC
Melanoma
MF
density
2.0
1.5
1.0
0.5

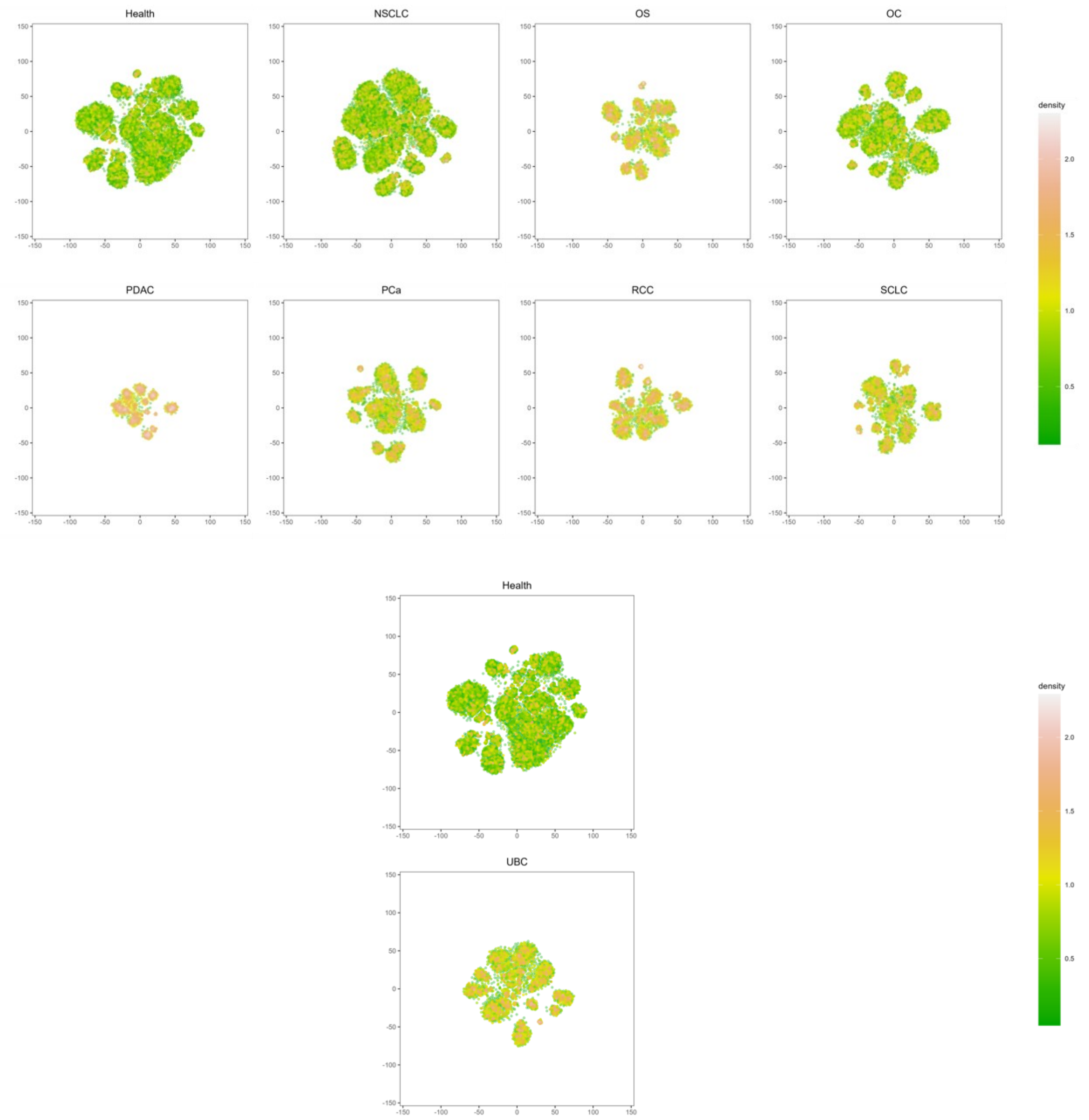

Fig. S2. Sample characteristic difference heat map. A pale yellow colour indicates a high density value, representing high-density regions of the TCR sequence. There is a higher concentration of TCR sequence points in these regions, which may indicate an increase in TCR sequence diversity in the cancer state. A green colour indicates a low density value, showing a lower density of TCR sequences. There are fewer TCR sequence points in these regions, which may reflect the stability of the TCR sequence in this state.

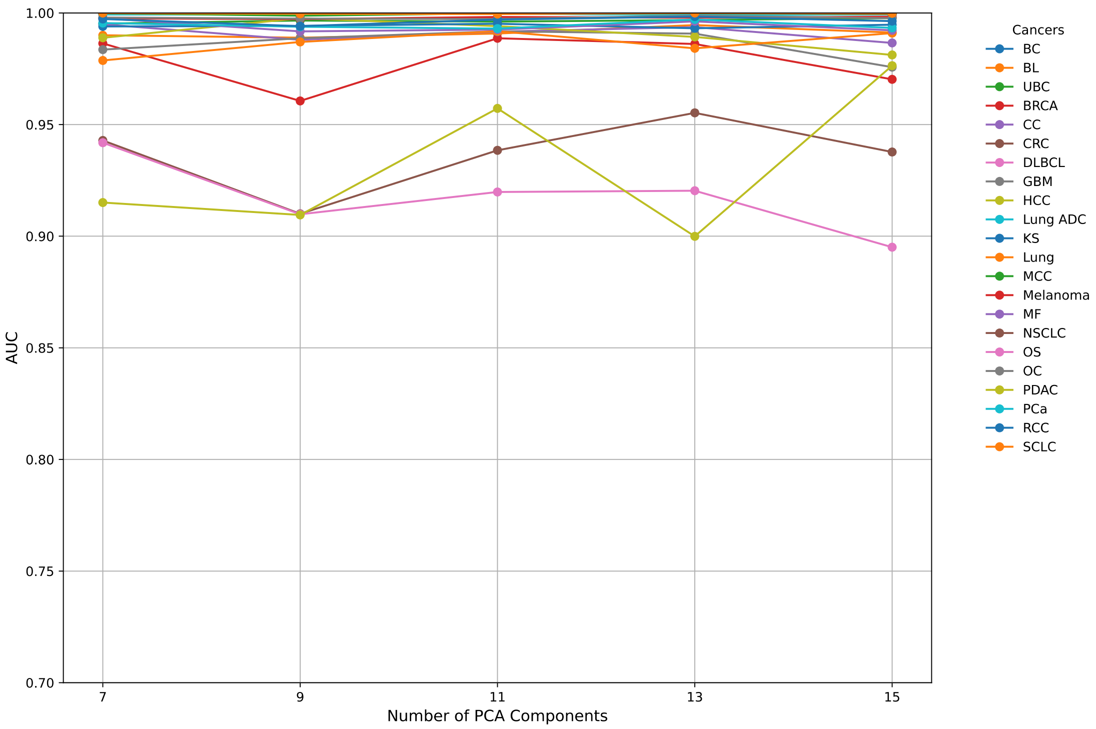

Fig. S3. Ablation experiment – number of PCA. This figure shows the impact of the first 7, 9, 11, 13 and 15 principal components on the model performance. The data are the 22 cancer datasets in this experiment, which are represented by lines of different colours in the figure. Each point represents the average AUC value of the 10 rounds of 5-fold cross-validation of the model with different numbers of principal components.

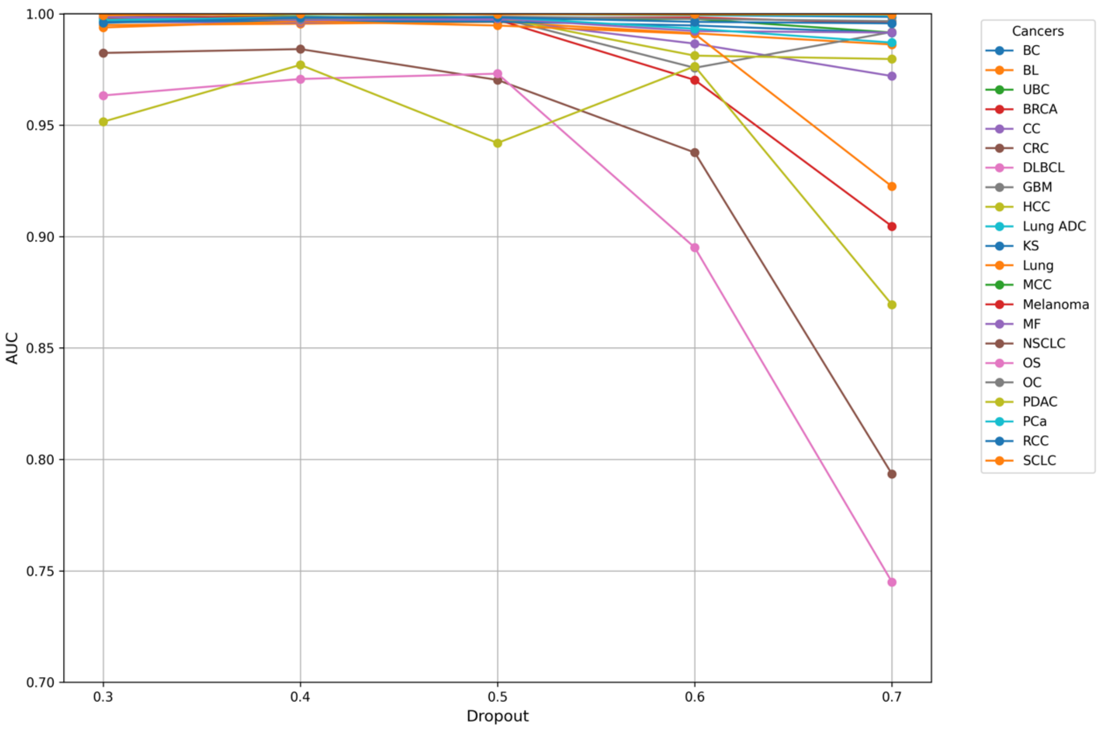

Fig. S4. Ablation experiment – Dropout. This graph shows the performance of the model as a function of the dropout value while holding other parameters constant. The data are the 22 cancer datasets in this experiment, which are represented by lines of different colours in the graph. Each point is the average AUC value of 10 rounds of 5-fold cross-validation of the model at different dropout values.

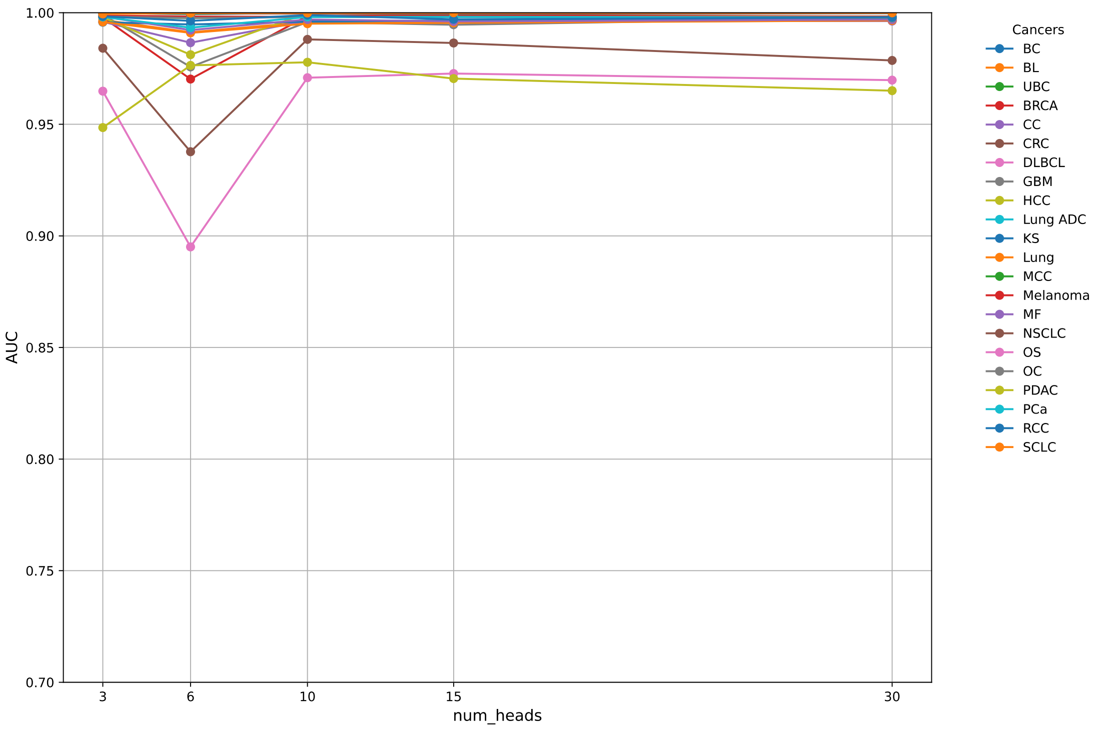

Fig. S5. Ablation experiment– number of attentions. This graph shows the performance of the model as a function of the number of attention heads while holding other parameters constant. The data are the 22 cancer datasets in this experiment, which are represented by lines of different colours in the graph. Each point represents the average AUC value of the 10-fold cross-validation of the model with different numbers of attention heads.

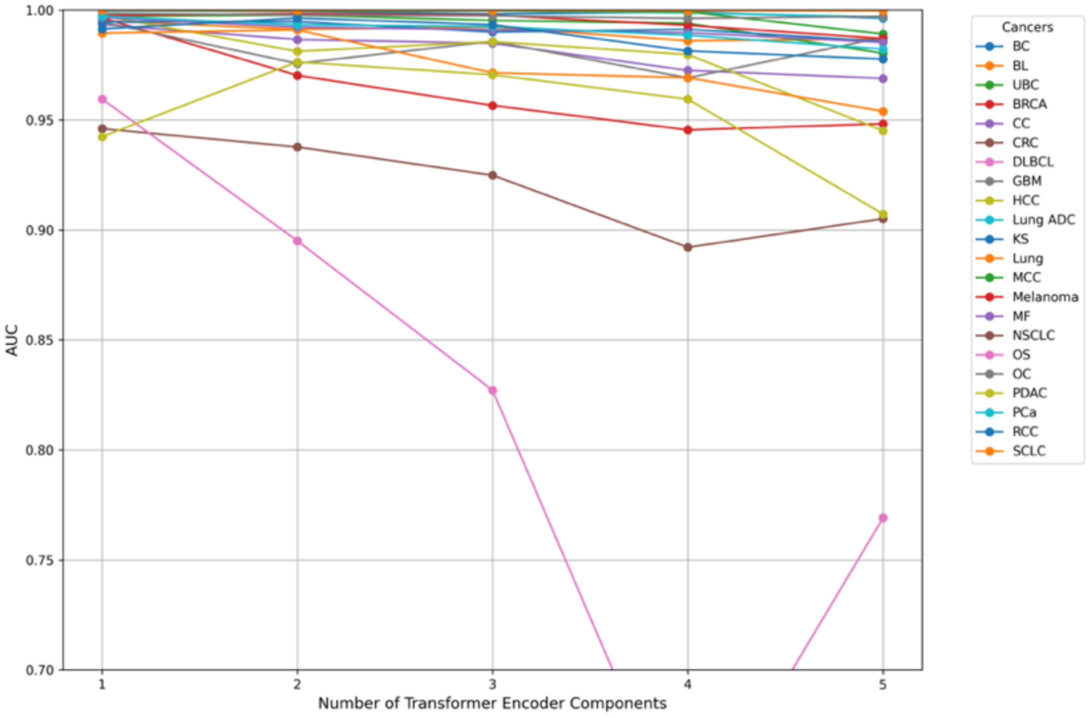

Fig. S6. Ablation experiment– number of encoders. This graph shows the performance of the model as a function of the number of encoders while holding other parameters constant. The data are the 22 cancer datasets in this experiment, represented by lines of different colours in the graph. Each point is the average AUC value of 10 rounds of 5-fold cross-validation for the model with different numbers of encoders.

Supplementary Tables

Table S1. Average performance of the models on the 22 cancer datasets.

Types Cancer	Model	ACC		SEN		SPE		AUC	
BC	**iCanTCR**	0.976921	0.005654	0.966667	0.00727	0.982383	0.006993	0.993433	0.002725
	**DeepLION**	0.973049	0.008433	0.964737	0.016207	0.977477	0.008695	0.99669	0.001394
	**DeepLION2**	0.94003	0.041285	0.839825	0.119988	0.993411	0.006198	0.993467	0.007282
	**MINN-SA**	0.734725	0.054431	0.673932	0.122076	0.76711	0.070388	0.826799	0.060798
	**TransMIL**	0.775868	0.029072	0.55467	0.070319	0.893703	0.031548	0.828767	0.03469
	**BiFormer**	0.975335	0.004709	0.959474	0.012202	0.983785	0.005731	0.995431	0.001301
	**AutoTFCNNY**	0.990274	0.006245	0.996316	0.005015	0.987056	0.009599	**0.99971**	**0.000247**
BL	**iCanTCR**	0.954613	0.007393	0.943957	0.011836	0.963925	0.009875	0.986909	0.003828
	**DeepLION**	0.926334	0.020427	0.912193	0.024526	0.938692	0.02042	0.979262	0.008388
	**DeepLION2**	0.930374	0.020995	0.887914	0.042719	0.967477	0.026715	0.982598	0.008535
	**MINN-SA**	0.749969	0.039892	0.695781	0.099872	0.797319	0.068318	0.833838	0.040779
	**TransMIL**	0.742966	0.028043	0.681899	0.042047	0.796328	0.035924	0.818026	0.026257
	**BiFormer**	0.929526	0.007566	0.914225	0.011525	0.942897	0.010408	0.97812	0.003242
	**AutoTFCNNY**	0.959302	0.010018	0.957326	0.010025	0.961028	0.019994	**0.994948**	**0.001806**
BRCA	**iCanTCR**	0.955023	0.007911	0.963864	0.010223	0.945935	0.013583	**0.991234**	**0.002899**
	**DeepLION**	0.876336	0.056283	0.881227	0.061937	0.871308	0.059083	0.951857	0.033521
	**DeepLION2**	0.938203	0.032889	0.9605	0.021551	0.91528	0.069892	0.988165	0.008372
	**MINN-SA**	0.702649	0.05965	0.871304	0.049808	0.529265	0.141687	0.788794	0.059172
	**TransMIL**	0.704697	0.024451	0.714417	0.039301	0.694704	0.032744	0.768063	0.026205
	**BiFormer**	0.929885	0.007087	0.942773	0.010258	0.916636	0.011987	0.979484	0.003079
	**AutoTFCNNY**	0.90841	0.039392	0.981955	0.032001	0.832804	0.070109	0.985651	0.013108
CC	**iCanTCR**	0.94652	0.007105	0.938359	0.015856	0.951402	0.010353	0.981747	0.003895
	**DeepLION**	0.89693	0.027806	0.843594	0.047215	0.928832	0.022345	0.959941	0.016221
	**DeepLION2**	0.90345	0.038321	0.780703	0.104393	0.976869	0.01365	0.974397	0.015467
	**MINN-SA**	0.782887	0.037527	0.653488	0.128083	0.860285	0.04617	0.853912	0.044179
	**TransMIL**	0.718944	0.028257	0.482165	0.071959	0.860568	0.031437	0.761838	0.039334
	**BiFormer**	0.91386	0.009408	0.865234	0.018055	0.942944	0.01104	0.968807	0.003758
	**AutoTFCNNY**	0.956959	0.01355	0.946484	0.022956	0.963224	0.019665	**0.994024**	**0.00271**
CRC	**iCanTCR**	0.942036	0.00982	0.91	0.018196	0.96	0.011356	**0.977921**	**0.005329**

	**DeepLION**	0.83015	0.028962	0.725583	0.056149	0.888785	0.020728	0.9031	0.027436
	**DeepLION2**	0.880419	0.030654	0.735833	0.090798	0.961495	0.02354	0.953512	0.016889
	**MINN-SA**	0.677312	0.046563	0.492088	0.115036	0.781176	0.109418	0.707248	0.047368
	**TransMIL**	0.691617	0.02682	0.364815	0.076047	0.87487	0.035409	0.708677	0.037861
	**BiFormer**	0.89012	0.013182	0.828917	0.027764	0.924439	0.015844	0.95322	0.007282
	**AutoTFCNNY**	0.856707	0.049791	0.869333	0.096748	0.849626	0.081202	0.948664	0.026823
**DLBCL**	**iCanTCR**	0.990556	0.005253	0.977609	0.012909	0.996121	0.003988	0.998698	0.001823
	**DeepLION**	0.99402	0.0036	0.982283	0.011422	0.999065	0.002203	0.999846	0.000281
	**DeepLION2**	0.944641	0.033404	0.81663	0.111809	0.999673	0.003271	0.999416	0.001069
	**MINN-SA**	0.922559	0.021171	0.814119	0.076946	0.969178	0.008804	0.96853	0.014501
	**TransMIL**	0.847792	0.021992	0.648112	0.063124	0.933635	0.020115	0.900772	0.025203
	**BiFormer**	0.99415	0.002985	0.981087	0.010086	0.999766	0.001024	0.999956	0.000073
	**AutoTFCNNY**	0.999771	0.000838	0.999239	0.002787	1	0	**1**	**0**
**GBM**	**iCanTCR**	0.956183	0.009742	0.843958	0.053832	0.981355	0.009567	0.983398	0.0089
	**DeepLION**	0.949313	0.014395	0.783542	0.069248	0.986495	0.007363	0.982869	0.009043
	**DeepLION2**	0.914084	0.020207	0.53125	0.110458	0.999953	0.000467	0.985333	0.016138
	**MINN-SA**	0.852456	0.017837	0.246212	0.116618	0.988436	0.01237	0.751443	0.085423
	**TransMIL**	0.835801	0.017104	0.302189	0.084146	0.955489	0.014431	0.761747	0.043987
	**BiFormer**	0.973511	0.006901	0.92	0.031158	0.985514	0.006457	**0.995438**	**0.00184**
	**AutoTFCNNY**	0.971565	0.0276	0.913542	0.076037	0.984579	0.02786	0.9923	0.011265
**HCC**	**iCanTCR**	0.925444	0.013724	0.803571	0.051204	0.957336	0.0118	0.970411	0.010378
	**DeepLION**	0.920407	0.01963	0.744286	0.080857	0.966495	0.009964	0.962926	0.018817
	**DeepLION2**	0.919222	0.015172	0.618393	0.0742	0.997944	0.004617	0.988019	0.010116
	**MINN-SA**	0.794126	0.009568	0.052128	0.051634	0.988294	0.01457	0.636972	0.069503
	**TransMIL**	0.830228	0.021195	0.39899	0.077928	0.943076	0.021809	0.748404	0.042129
	**BiFormer**	0.934037	0.0099	0.793036	0.040139	0.970935	0.007705	0.979302	0.004104
	**AutoTFCNNY**	0.96463	0.021033	0.928036	0.069576	0.974206	0.023213	**0.993783**	**0.008922**
**KS**	**iCanTCR**	0.962318	0.006123	0.973898	0.006097	0.936776	0.015696	0.992607	0.002035
	**DeepLION**	0.935496	0.009963	0.966949	0.008103	0.866121	0.02754	0.97891	0.004658
	**DeepLION2**	0.93188	0.017193	0.984661	0.00666	0.815467	0.056761	0.98514	0.005984
	**MINN-SA**	0.75737	0.020502	0.896037	0.029834	0.451525	0.090149	0.787147	0.027035
	**TransMIL**	0.747769	0.019209	0.897342	0.021053	0.41787	0.054691	0.769145	0.026709

	**BiFormer**	0.932536	0.007039	0.962669	0.006637	0.866075	0.017738	0.976441	0.003805
	**AutoTFCNNY**	0.965364	0.00638	0.986631	0.005018	0.918458	0.021536	**0.995308**	**0.001112**
**Lung**	**iCanTCR**	0.937877	0.010197	0.88964	0.020543	0.962897	0.010498	0.979515	0.0054
	**DeepLION**	0.903938	0.021248	0.834865	0.049643	0.939766	0.015404	0.963606	0.014317
	**DeepLION2**	0.921446	0.030727	0.802072	0.092987	0.983364	0.01116	0.978812	0.010689
	**MINN-SA**	0.65296	0.069949	0.566294	0.126735	0.697914	0.108396	0.695223	0.087395
	**TransMIL**	0.739052	0.025892	0.491491	0.067359	0.86746	0.030243	0.757057	0.034992
	**BiFormer**	0.923015	0.009818	0.868018	0.023554	0.951542	0.009754	0.978922	0.003761
	**AutoTFCNNY**	0.942369	0.030296	0.941441	0.0518	0.94285	0.043263	**0.990132**	**0.007699**
**Lung ADC**	**iCanTCR**	0.985618	0.004032	0.988165	0.005665	0.983738	0.005536	0.998883	0.000612
	**DeepLION**	0.969892	0.023099	0.973671	0.028071	0.967103	0.022853	0.995809	0.006594
	**DeepLION2**	0.978575	0.011522	0.972215	0.025042	0.983271	0.009183	0.998151	0.001632
	**MINN-SA**	0.791273	0.038415	0.721263	0.106495	0.842962	0.039969	0.875815	0.032841
	**TransMIL**	0.78405	0.025978	0.722606	0.041798	0.829416	0.031477	0.849089	0.027484
	**BiFormer**	0.977446	0.004535	0.985759	0.007706	0.971308	0.006576	0.997332	0.000874
	**AutoTFCNNY**	0.983468	0.010494	0.995506	0.005191	0.974579	0.018132	**0.999621**	**0.000362**
**MCC**	**iCanTCR**	0.988951	0.005346	0.979722	0.014828	0.992056	0.004812	0.998709	0.001193
	**DeepLION**	0.981783	0.007941	0.957083	0.021902	0.990093	0.006911	0.998168	0.001409
	**DeepLION2**	0.945594	0.030394	0.788333	0.118972	0.998505	0.006834	0.998124	0.005281
	**MINN-SA**	0.839797	0.021895	0.510662	0.110682	0.950533	0.017999	0.875616	0.040165
	**TransMIL**	0.800381	0.025015	0.371352	0.085354	0.944728	0.023096	0.795586	0.045402
	**BiFormer**	0.986294	0.006826	0.98125	0.015339	0.987991	0.007068	0.999173	0.000778
	**AutoTFCNNY**	0.996189	0.00419	0.99625	0.006197	0.996168	0.005492	**0.999952**	**0.000091**
**MF**	**iCanTCR**	0.949968	0.008473	0.911979	0.021475	0.967009	0.009735	0.984308	0.00622
	**DeepLION**	0.956355	0.009446	0.908646	0.021903	0.977757	0.007973	0.988909	0.003475
	**DeepLION2**	0.906097	0.034317	0.704792	0.114284	0.996402	0.007149	0.98508	0.007199
	**MINN-SA**	0.827142	0.026906	0.612269	0.104851	0.923534	0.024368	0.884574	0.029112
	**TransMIL**	0.771522	0.025642	0.44455	0.077321	0.918201	0.024549	0.802808	0.038572
	**BiFormer**	0.963484	0.005905	0.921771	0.011029	0.982196	0.006565	0.987092	0.002606
	**AutoTFCNNY**	0.971129	0.006259	0.915937	0.018566	0.995888	0.005839	**0.995937**	**0.001804**
**Melanoma**	**iCanTCR**	0.958323	0.007067	0.967046	0.008623	0.946869	0.015571	0.993631	0.001905
	**DeepLION**	0.945434	0.011562	0.950712	0.0129	0.938505	0.014862	0.988294	0.003822

	**DeepLION2**	0.953636	0.016312	0.966833	0.015614	0.936308	0.038726	0.992386	0.003088
	**MINN-SA**	0.74927	0.060141	0.922032	0.03783	0.52242	0.156781	0.842883	0.062288
	**TransMIL**	0.767105	0.027965	0.814336	0.030912	0.705088	0.041388	0.841306	0.027121
	**BiFormer**	0.940545	0.006643	0.954698	0.007345	0.921963	0.013876	0.986392	0.002656
	**AutoTFCNNY**	0.975879	0.007481	0.988399	0.004485	0.959439	0.015869	**0.998096**	**0.000773**
**NSCLC**	**iCanTCR**	0.980137	0.006842	0.984107	0.005543	0.975981	0.012759	0.995488	0.002611
	**DeepLION**	0.984726	0.005388	0.985759	0.007198	0.983645	0.008202	0.999117	0.000473
	**DeepLION2**	0.962785	0.035629	0.969509	0.040862	0.955748	0.05126	0.994628	0.010261
	**MINN-SA**	0.883077	0.037131	0.912653	0.044438	0.852119	0.056346	0.960191	0.021011
	**TransMIL**	0.80619	0.031848	0.818317	0.038129	0.793496	0.038172	0.887424	0.030273
	**BiFormer**	0.978402	0.003663	0.980446	0.006079	0.976262	0.006359	0.998303	0.000517
	**AutoTFCNNY**	0.994018	0.003023	0.998571	0.002187	0.989252	0.006141	**0.999929**	**0.000122**
**OC**	**iCanTCR**	0.954437	0.007735	0.954652	0.012098	0.954206	0.016119	0.988358	0.00322
	**DeepLION**	0.949077	0.023478	0.953348	0.022763	0.944486	0.02836	0.990771	0.008284
	**DeepLION2**	0.9375	0.0438	0.947174	0.042372	0.927103	0.08019	0.9871	0.016085
	**MINN-SA**	0.771726	0.045883	0.894862	0.046086	0.639384	0.098145	0.865436	0.039784
	**TransMIL**	0.765152	0.027017	0.76043	0.034841	0.770226	0.036934	0.843796	0.025298
	**BiFormer**	0.948649	0.005894	0.941826	0.009347	0.955981	0.011603	0.990887	0.001891
	**AutoTFCNNY**	0.974369	0.007164	0.984043	0.005699	0.963972	0.015004	**0.998194**	**0.000782**
**OS**	**iCanTCR**	0.943059	0.0107	0.803415	0.061705	0.969813	0.006522	**0.953319**	**0.012294**
	**DeepLION**	0.869412	0.014379	0.288537	0.086323	0.980701	0.008651	0.835986	0.045572
	**DeepLION2**	0.87051	0.010277	0.195122	0.064484	0.999907	0.000658	0.925323	0.032737
	**MINN-SA**	0.861596	0.00872	0.183543	0.060183	0.991504	0.007045	0.715094	0.080271
	**TransMIL**	0.83775	0.009533	0.044592	0.041672	0.98971	0.010341	0.66112	0.051742
	**BiFormer**	0.910078	0.010711	0.606585	0.050554	0.968224	0.009486	0.912717	0.011602
	**AutoTFCNNY**	0.93149	0.029013	0.701951	0.098088	0.975467	0.031855	0.952545	0.02974
**PCa**	**iCanTCR**	0.941883	0.010768	0.907872	0.035468	0.956822	0.016896	0.984323	0.004284
	**DeepLION**	0.927825	0.017111	0.864681	0.035107	0.955561	0.014844	0.978568	0.008722
	**DeepLION2**	0.822792	0.047834	0.440957	0.164984	0.990514	0.010406	0.930232	0.043495
	**MINN-SA**	0.75856	0.031157	0.337847	0.131282	0.943359	0.035313	0.778543	0.073159
	**TransMIL**	0.765479	0.021887	0.472276	0.056698	0.89427	0.026422	0.784345	0.030659
	**BiFormer**	0.933247	0.007446	0.895426	0.017766	0.94986	0.008424	0.980372	0.003751

	**AutoTFCNNY**	0.97276	0.010245	0.946277	0.025633	0.984393	0.009811	**0.996686**	**0.002799**
**PDAC**	**iCanTCR**	0.921217	0.01145	0.121875	0.067872	0.980981	0.01428	0.75446	0.071125
	**DeepLION**	0.93113	0.00348	0.0225	0.040241	0.999065	0.002301	0.789355	0.052129
	**DeepLION2**	0.930478	0.000435	0.000625	0.00625	1	0	0.771043	0.082504
	**MINN-SA**	0.930435	0	0	0	1	0	0.47994	0.050361
	**TransMIL**	0.922354	0.010989	0.087753	0.068535	0.984754	0.010961	0.514154	0.08095
	**BiFormer**	0.940739	0.007303	0.23125	0.109341	0.993785	0.005274	0.853645	0.060145
	**AutoTFCNNY**	0.946478	0.010167	0.261875	0.145344	0.997664	0.004279	**0.94965**	**0.033732**
**RCC**	**iCanTCR**	0.948202	0.012799	0.877736	0.044753	0.965654	0.012626	0.974588	0.011604
	**DeepLION**	0.94397	0.01208	0.792453	0.056253	0.981495	0.006803	0.973557	0.011453
	**DeepLION2**	0.914082	0.020997	0.568113	0.106831	0.999766	0.001922	0.987377	0.011608
	**MINN-SA**	0.839973	0.023346	0.26701	0.149161	0.981875	0.01312	0.79351	0.083712
	**TransMIL**	0.847312	0.018487	0.390128	0.086498	0.96054	0.018454	0.792349	0.037457
	**BiFormer**	0.949775	0.010199	0.831321	0.048481	0.979112	0.006657	0.978774	0.006761
	**AutoTFCNNY**	0.975918	0.010174	0.905283	0.042739	0.993411	0.007125	**0.997364**	**0.00398**
**SCLC**	**iCanTCR**	0.984887	0.004867	0.951346	0.022449	0.993037	0.00443	0.993839	0.004481
	**DeepLION**	0.975639	0.009932	0.899615	0.044292	0.994112	0.004998	0.996756	0.002537
	**DeepLION2**	0.918383	0.027132	0.583846	0.138554	0.999673	0.001198	0.994047	0.01396
	**MINN-SA**	0.866105	0.022701	0.422883	0.132453	0.973803	0.01275	0.860526	0.068082
	**TransMIL**	0.833333	0.018093	0.315657	0.083102	0.959124	0.017565	0.791513	0.047462
	**BiFormer**	0.983872	0.005077	0.949615	0.02131	0.992196	0.003932	0.997918	0.001142
	**AutoTFCNNY**	0.99688	0.004781	0.993269	0.01456	0.997757	0.004625	**0.999933**	**0.0002**
**UBC**	**iCanTCR**	0.953293	0.00896	0.932735	0.01784	0.964533	0.01288	0.9878	0.00409
	**DeepLION**	0.913988	0.025651	0.857863	0.054905	0.944673	0.018222	0.972939	0.014348
	**DeepLION2**	0.930544	0.030877	0.83	0.090322	0.985514	0.011964	0.986713	0.008951
	**MINN-SA**	0.72236	0.046421	0.533886	0.165622	0.825404	0.074493	0.769264	0.072773
	**TransMIL**	0.776343	0.023939	0.608996	0.054445	0.867837	0.028837	0.818024	0.029568
	**BiFormer**	0.942054	0.009372	0.924188	0.020782	0.951822	0.010347	0.985136	0.003949
	**AutoTFCNNY**	0.972024	0.011145	0.943675	0.030421	0.987523	0.009323	**0.997517**	**0.001447**

The maximum AUC values for the evaluation metrics in the comparison models are shown in bold. The values in parentheses represent standard deviations. BC: Brain Cancer; BL: Burkitt Lymphoma; BRCA: Breast Cancer; CC: Cervical Cancer ; CRC: Colorectal Cancer; DLBCL: Lymphoid Neoplasm Diffuse Large B-cell Lymphoma; GBM: Glioblastoma Multiforme; HCC: Hepatocellular Carcinoma;

KS: Kaposi sarcoma; Lung: Lung Cancer; Lung ADC: Lung Adenocarcinomas; MCC: Merkel Cell Carcinoma; MF: Mycosis fungoides; Melanoma; NSCLC: Non-small Cell Lung Cancer; OC: Ovarian Cancer; OS: Osteosarcoma; PCa: Prostate Cancer; PDAC: Pancreatic Ductal Adenocarcinoma; RCC: Renal Cell Carcinoma; SCLC: Small-cell Lung Cancer; UBC: Urothelial Bladder Cancer; ACC, accuracy; SEN, sensitivity; SPE, specificity; AUC, area under the receiver operating characteristic curve.

Table S2. Summary information of experimental dataset.

Disease	Total sample size	Sample size	Data type	LOCUS	Study	immuneACCESS DOI or DOI
**BC**	114	144	TCR-seq	TCRB	Kudo Y et al.	https://doi.org/10.21417/YK2019AO
**BL**	187	153	TCR-seq	IGKLIGHTCRB	K Lombardo et al.,	https://doi.org/10.21417/B7MS37
		115	TCR-seq	TCRB	J Rieken et al.	https://doi.org/10.21417/JR20202BJH
**BRCA**	220	115	TCR-seq	TCRB	DB Page et al.	https://doi.org/10.21417/DBP2016CIR
		107	TCR-seq	TCRB	Page DB et al.	https://doi.org/10.21417/DBP2023NPJ
**CC**	128	128	TCR-seq	TCRB	L Colbert et al.	https://doi.org/10.21417/LC2023CC
**CRC**	120	92	TCR-seq	TCRB	Høye E et al.	https://doi.org/10.21417/EH2023GS
		28	TCR-seq	TCRB	AM Sherwood et al.	https://doi.org/10.21417/B7PP46
**DLBCL**	92	92	TCR-seq	TCRB	C Keane et al.	https://doi.org/10.21417/B7C301
**GBM**	48	48	TCR-seq	TCRB	MS Hsu et al.	https://doi.org/10.21417/B7D59F
**HCC**	56	56	TCR-seq	TCRB	M Yarchoan et al.	https://doi.org/10.21417/RP2024NM
**KS**	472	474	TCR-seq	TCRB	S Ravishankar et al.	https://doi.org/10.21417/SR2024JEM

**Lung**	111	40	TCR-seq	TCRB	AS Mansfield et al.	https://doi.org/10.21417/B71P7F
		29	TCR-seq	TCRB	JX Caushi et al.	https://doi.org/10.21417/JC2021N
		22	TCR-seq	TCRB	D Hamm	https://doi.org/10.21417/ADPT2020V4CD
		20	TCR-seq	TCRB	SC Formenti et al.	https://doi.org/10.21417/B7BW6X
**Lung ADC**	158	158	TCR-seq	TCRB	H. Dejima et al.	https://doi.org/10.1038/s41467-021-22890-x
**MCC**	72	72	TCR-seq	TCRB	M Farah et al.	https://doi.org/10.21417/MF2020JID
**MF**	96	68	TCR-seq	TCRB	D. Joffe et al.	https://doi.org/10.21417/DJ2023BA
		28	TCR-seq	TCRB	Z Yu et al.	https://doi.org/10.21417/RC2023JID
**Melanoma**	281	199	TCR-seq	TCRB	W Pruessmann et al.	https://doi.org/10.21417/WP2019NC
		84	TCR-seq	TCRB	Huuhtanen J et al.	https://doi.org/10.21417/JH2023JCI
**NSCLC**	224	224	TCR-seq	TCRB	A Reuben et al.	https://doi.org/10.21417/AR2019NC
**OC**	230	96	TCR-seq	TCRB	Ryan O Emerson et al.	https://doi.org/10.21417/B7TG64
		90	TCR-seq	TCRB	s Lee; L Zhao et al.	https://doi.org/10.21417/LS2021iS
		44	TCR-seq	TCRB	K Yoshida-Court et al.	https://doi.org/10.21417/KC2023PO
**OS**	41	41	TCR-seq	TCRB	C Wu et al.	https://doi.org/10.21417/CW2020NC
**PCa**	94	94	TCR-seq	TCRB	E Shenderov et al.	https://doi.org/10.21417/ES2023NM
**PDAC**	16	16	TCR-seq	TCRB	Stromnes I et al.	https://doi.org/10.21417/B7305D
**RCC**	53	53	TCR-seq	TCRB	J Chow et al.	https://doi.org/10.21417/JC2020PNAS
**SCLC**	52	52	TCR-seq	TCRB	M Chen et al.	https://doi.org/10.21417/MC2021NC
**UBC**	117	117	TCR-seq	TCRB	A Snyder et al.	https://doi.org/10.21417/B7MG68
**Health**	214	214	TCR-seq	TCRB	Ying Xu et al.	https://doi.org/10.3389/fgene.2022.860510

BC: Brain Cancer; BL: Burkitt Lymphoma; BRCA: Breast Cancer; CC: Cervical Cancer ; CRC: Colorectal Cancer; DLBCL: Lymphoid Neoplasm Diffuse Large B-cell Lymphoma; GBM: Glioblastoma Multiforme; HCC: Hepatocellular Carcinoma; KS: Kaposi sarcoma; Lung: Lung Cancer; Lung ADC: Lung Adenocarcinomas; MCC: Merkel Cell Carcinoma; MF: Mycosis fungoides; Melanoma; NSCLC: Non-small Cell Lung Cancer; OC: Ovarian Cancer; OS: Osteosarcoma; PCa: Prostate Cancer; PDAC: Pancreatic Ductal Adenocarcinoma; RCC: Renal Cell Carcinoma; SCLC: Small-cell Lung Cancer; UBC: Urothelial Bladder Cancer; Health： Non-cancer; T Cell Receptor-sequencing.

Table S3. Summary information of test datasets.

Disease	Sample size	Data type	LOCUS	Study	immuneACCESS DOI or DOI
**UBC**	12	TCR-seq	TCRB	Sankin A et al.	https://doi.org/10.21417/AS2019UO
**BRCA**	23	TCR-seq	TCRB	Muraro E et al.	https://doi.org/10.21417/EM2022FO
**NSCLC**	66	TCR-seq	TCRB	Kargl J et al.	https://doi.org/10.21417/B7B88G
**Melanoma**	21	TCR-seq	TCRB	Huuhtanen J - et al.	https://doi.org/10.21417/JH2022NC
**CRC**	10	TCR-seq	TCRB	Rajamanickam V et al.	https://doi.org/10.21417/vr2021cir
**Health**	51	TCR-seq	TCRB	Ying Xu et al.	https://doi.org/10.3389/fgene.2022.860510

BRCA: Breast Cancer; CRC: Colorectal Cancer; Melanoma; NSCLC: Non-small Cell Lung Cancer; UBC: Urothelial Bladder Cancer; TCR-seq: Health： Non-cancer; T Cell Receptor-sequencing.
